# Unassuming Lichens: Nature’s Hidden Antimicrobial Warriors

**DOI:** 10.3390/ijms26073136

**Published:** 2025-03-28

**Authors:** Hongqiao Tian, Junlin Lu, Fangrong Liang, Haiyan Ding, Chaojiang Xiao

**Affiliations:** 1 College of Public Health, Dali University, Dali 671003, China; 18314368561@163.com (H.T.);; 2 College of Pharmacy, Dali University, Dali 671003, China

**Keywords:** antimicrobial activity, lichens, drug-resistant microorganisms, pharmaceutical field

## Abstract

In a hidden corner of the Earth, an ongoing war is being waged: a battle between lichens and microorganisms. Lichens, ancient and unique symbiotic organisms, with their unique survival wisdom, are bursting with vitality in extreme environments. Over 80% of secondary metabolites in lichens are not found in other organisms, making lichen-derived compounds a promising resource for the development of new drugs, particularly against drug-resistant microorganisms, due to their distinctive chemical structures and biological activities. This article aims to explore in depth the lichen species exhibiting antimicrobial activity and their antimicrobial metabolites and focus on unique compounds such as divaricatic acid, usnic acid, vulpinic acid, salazinic acid, and rhizocarpic acid, which demonstrate significant antimicrobial effects against various resistant microorganisms, including methicillin-resistant *Staphylococcus aureus*, drug-resistant *Mycobacterium tuberculosis*, and *Candida albicans* and other drug-resistant microorganisms. Meanwhile, this paper discusses the potential applications and challenges associated with the use of lichens in medicine, agriculture, and food industry, aiming to elucidate these mysterious organisms for lichen researchers and enthusiasts while promoting further research and applications in the field of antimicrobials.

## 1. Introduction

**Figure 1:**
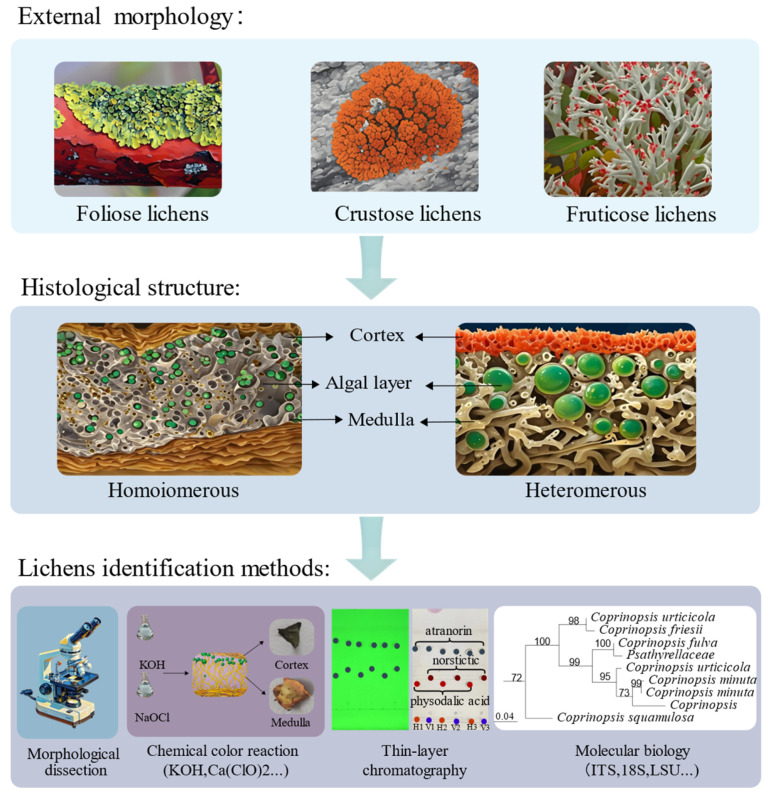
The fundamental structure and biological identification methods of lichens.

Lichens, symbiotic organisms composed of fungi and algae [1], exhibit an extremely broad ecological distribution on Earth [2], ranging from the equator to the poles and from the Gobi Desert to high-altitude tundra and volcanic islands, even thriving in extreme environments close to space. They are found on nearly all terrestrial surfaces [3,4], covering approximately 8% of the planet’s land area [5], and play crucial roles in ecosystems as “pioneers” [4]. Based on external morphology, lichens can be classified into crustose lichens, foliose lichens, and fruticose lichens [4]. From an internal structural perspective, lichens can be divided into homoiomerous thalli and heteromerous thalli. Homoiomerous lichens have algal cells evenly distributed within the mycelial tissue without obvious layering, while heteromerous lichens exhibit a distinct layered structure. In the classification and identification of lichens, morphological features, chemical analyses, and molecular biology methods are usually integrated [4]. Common identification methods include morphological dissection, chemical color reaction methods, thin-layer chromatography, and molecular biology techniques [4]. The combined application of these methods helps accurately identify lichens, providing solid scientific evidence for related research (Figure 1).

Although more than 26,000 species of lichens are currently known worldwide, they receive far less attention in the scientific community compared with many other organisms [6,7]. Nonetheless, the chemical diversity and substantial biological activity of lichens present great potential for the development of novel antimicrobial applications. Lichens produce a wide array of structurally unique secondary metabolites [3], demonstrating high efficacy against bacterial, fungal, and viral pathogens, thus representing valuable resources for developing new strategies against drug-resistant microorganisms [8]. This antimicrobial potential is closely related to the defense mechanisms, niche competition, self-protective abilities, adaptive evolution, and biodiversity of lichens. Specifically, the antimicrobial compounds in lichens are believed to be evolutionary products of their defense mechanisms, primarily functioning to prevent microbial colonization and competition [1]. These compounds effectively inhibit the growth of bacteria, fungi, and other microorganisms, helping lichens survive in diverse and nutrient-poor environments [9]. Additionally, lichens typically grow in ecological niches with limited resources, such as rocky surfaces or tree barks [4]. By producing antimicrobial substances, lichens can reduce competition from other microorganisms, enhancing their survival capabilities, which facilitates more effective habitation and reproduction [2]. Some antimicrobial compounds also serve to protect their photosynthetic partners from Ultraviolet (UV) radiation or desiccation [10]. This multifunctionality allows lichens to thrive in extreme environments, further enhancing their adaptability [9]. At the same time, there is a complex interaction between lichens and the microbial communities in their environment. Certain lichens may evolve specific antimicrobial compounds in response to changes in local microbial ecology, forming a co-evolutionary dynamic [11]. This dynamic not only influences the ecological roles of lichens but also enriches the diversity of their chemical compounds [12,13]. Finally, the diversity of lichen species and their various growth environments enable them to produce a wide range of antimicrobial compounds. This chemical diversity reflects the important ecological roles of lichens in their ecosystems and may provide new avenues for drug development and the discovery of natural antimicrobial agents [14].

Despite the considerable antimicrobial potential of lichens, research in this area reveals several gaps. Most studies on antimicrobial activity primarily focus on specific species or compounds, resulting in a lack of comprehensive overviews of their biodiversity and the underlying active substances. Additionally, the potential applications of lichens in medicine, agriculture, and the food industry remain insufficiently explored, particularly regarding their role in combating antibiotic resistance, an area where research is still in its preliminary stages [15]. Moreover, the unclear taxonomy of some lichens, slow growth rate restricting raw material availability [4], technical challenges involved in cultivation [16], limitations in compound isolation and purification techniques [17], and uncertain potential adverse reactions and toxicity [18,19] have hindered in-depth research and widespread application of lichen resources. In response to identified research gaps, this paper systematically reviews the potential of lichens as natural antimicrobial resources, providing an in-depth discussion on existing issues in the field. It analyzes the biodiversity of antimicrobial lichens and their active substance basis, explores their application prospects for addressing drug-resistant infections, and presents new insights for drug development and microbiological research. The authors call on the academic community to enhance research efforts on lichens to fully realize their potential value in medicine, agriculture, and the food industry. Additionally, this study highlights current challenges, including taxonomic ambiguity, technical bottlenecks, and potential toxicity, while proposing future research directions to combat microbial resistance. The references cited in this article are primarily sourced from the following databases: Web of Science, PubMed, and Google Scholar, where the keywords used included “lichens, *Parmeliaceae*, *Ramalinaceae*, *Usnea* spp., antimicrobial activity, antibacterial activity, antifungal activity, antiviral activity, secondary metabolites, medical applications, agricultural applications, food industry”, along with related synonyms and combinations. In the literature selection process, we prioritized peer-reviewed articles published in the last decade, focusing on research related to the antimicrobial activity of lichens or lichen-derived compounds and their potential application challenges, while excluding duplicates. Through systematic screening and analysis of the literature, we summarize research progress on the antimicrobial activity of lichens, discuss their application potential across various fields, and identify existing challenges as well as future development directions.

## 2. Types and Characteristics of Antimicrobially Active Lichens

Globally, there are as many as 26,000 lichen species distributed in 500 genera [20], and 109 lichens exhibiting significant antimicrobial activity have been reported in the current literature, involving 41 genera and 20 families. Among these, lichens from the family *Parmeliaceae* dominated the antimicrobial active lichens, representing 44%, the family *Ramalinaceae* accounts for 15%, and the remaining 41% of the active lichens were distributed in 18 families such as *Cladoniaceae*, *Eloschistaceae*, *Caliciaceae*, etc. (Table 1). Among these active lichens, lichens of the genus *Usnea* in the family *Parmeliaceae* [21,22,23] are particularly prominent due to their abundance of species and diverse bioactivities; e.g., lichens such as *Usnea steineri* [24] and *Usnea articulate* [25] exhibit excellent antimicrobial effects. In addition, lichens from the genera *Parmelia* [26,27], *Lobaria* [28,29], and *Thamnolia* [28] have also shown significant antimicrobial activity and may be potential antibiotic or pesticide candidates.

At the level of antimicrobial targets, lichen extract exhibits significant antimicrobial activity against a wide range of pathogenic microorganisms, covering Gram-positive, Gram-negative, and clinically resistant bacteria, as well as plant and animal pathogenic fungi. In terms of against Gram-positive bacteria, lichen extracts of *Usnea barbata* [21], *Parmelia perlata* [26], *Cladonia foliacea* [30], and *Cryptothecia striata* [31] against *Bacillus subtilis* [21,32], *Streptococcus pneumonia* [21], *Staphylococcus epidermidis* [24], *Staphylococcus aureus* [29,32,33], *Lactobacillus plantarum* [34], *Enterococcus* spp. [22,35], *Listeria* spp. [30,36], and *Micrococcus luteus* [31] are potential pathogens that exhibit antimicrobial activity, and these strains are commonly associated with hospital-acquired infections, highlighting the potential of antimicrobial activity of lichens for medical applications. Against Gram-negative bacteria, lichens extract effectively inhibited highly pathogenic strains such as *Salmonella typhi* [25,37], *Vibrio cholerae* [31,32], *Aeromonas hydrophila* [30], and *Pseudomonas aeruginosa* [22,38], as well as *Enterobacter* [39], *Escherichia* [25,26], *Proteus* [26,33], *Citrobacter* [25] and *Vibrio* [31,32] bacteria. Against clinically resistant bacteria, lichen extracts have also shown good antimicrobial activity against resistant strains such as methicillin-resistant *Staphylococcus aureus* [35,40], quinolone-resistant *Escherichia coli* [41], vancomycin-resistant *Enterococcus faecalis* [24], *Acinetobacter baumannii* [35], multidrug-resistant *Mycobacterium tuberculosis* (MDR-A8, MDR-V791) and *Mycobacterium smegmatis* (MDR-R, MDR-40) [23], which provides a new source of substances to cope with the increasing antibiotic resistance problem. Particularly in antituberculosis, lichens extract can effectively inhibit a variety of *Mycobacterium tuberculosis*, including *Mycobacterium tuberculosis* H37Ra, *Mycobacterium smegmatis* [23], *Mycobacterium tuberculosis*, *Mycobacterium kansasii*, and *Mycobacterium avium* [24] complex groups, and the high efficiency in against *Mycobacterium tuberculosis* has provided new ideas for the development of new antituberculosis drugs. In terms of antifungal activity, lichen extracts have also shown encouraging results against animal pathogenic fungi such as *Cryptococcus neoformans* [42], and *Candida* spp. such as *Candida albicans* [21,22,32], *Candida parapsilosis* [22], and *Candida glabrata* [30], as well as the phytopathogenic fungi *Cladosporium cladosporioides* [43], *Colletotrichum capsici* [44], *Fusarium oxysporum* [43,44,45], *Achlya bisexualis* [46], *Bipolaris sorokiniana* [44,46], and *Saprolegnia parasitica* [46], *Pythium debaryanum* [27], *Fusarium fujikuroi* [47], *Rhizoctonia solani* [27,39] and Dermatophyte [34], have demonstrated good antimicrobial effects and may be highly promising natural resources for the development of novel antifungal drugs. Lichen extracts have important applications in medicine and agriculture due to their wide range of antimicrobial activities and deserve further research and development.

While delving into the antimicrobial potential of lichens extract, it was noticed that the antimicrobial active components were mainly concentrated in the organic solvent extracts phases, especially in the solvent extract phases such as methanol, ethanol, ethyl acetate, and acetone. Among them, methanol [32,33] and acetone [48,49] extracts were able to capture more active components due to their good solubilization properties, which endowed the extracts with potent antimicrobial activities. However, this pattern is not invariable; for example, the ethyl acetate extract of *Parmelia reticulate* showed better antifungal activity than the methanol extract, suggesting that the variety of lichens actively enriched in different organic phases is rich and diverse [27]. In addition to the organic solvents mentioned above, dichloromethane [38], n-hexane [37,38], chloroform [30], acetonitrile [38], propyl alcohol [47], and oil [50] have also been widely used for the extraction of lichens compounds, providing diverse options for antimicrobial studies of lichens. Although aqueous extracts have antimicrobial activity in some specific cases, such as the inhibitory activity of *Parmelia cirrhatum* aqueous extracts against pathogenic fungi [27], the expression of this bioactivity is dependent on the properties of the compounds and is not a universal phenomenon. In most cases, lichen aqueous extracts lack antimicrobial activity; e.g., *Ramalina sinensi* [25] and *Usnea barbata* [21] aqueous extracts are not active against the target. Our previous study also further corroborated the advantages of organic solvent extracts in antimicrobial activity, which was especially evident in the screening of the antimicrobial activity of lichens from Cangshan Mountain, Dali, China [51]. Therefore, relevant researchers should pay special attention to this feature of active ingredients from lichens during the development process of exploring the development of active ingredients from lichens.

**Table 1:** Antimicrobial activity of lichens extracts.

Categories	Object Strain	Lichens (Extracts)	Sample	Positive Control	References
			MIC/MBC/ED50 (µg/mL)/IZ (mm)/IR (%)/RIZD (%)		
Gram-positive bacteria	*Bacillus subtilis*	*Usnea barbata* (*Methanol-acetone*)	MIC: 100		[21]
			IZ: 27.0		[52]
		*Usnea rubrotincta* (*Acetone*)	MIC: 15.63	Chloramphenicol (MIC: 7.81) Vancomycin (MIC: 7.81)	[53]
		*Usnea rubrotincta* (*Methanol*)	MIC: 250		
		*Parmelia conspersa* (*Methanol*)	MIC: 156.25	Amracin (MIC: 0.24)	[26]
		*Parmelia conspersa* (*Acetone*)	MIC: 39.1		
		*Parmelia perlata* (*Methanol*)	MIC: 78.125		
		*Parmelia perlata* (*Acetone*)	MIC: 126.25		
		*Parmelia sulcata* (*Acetone*)	IZ: 25.0		[27]
			MIC: 3120		
		*Evernia prunastri* (*Acetone*)	MIC: 78		[36]
		*Pseudever niafurfuracea* (*Acetone*)	MIC: 78		
		*Ramalina sinensis* (*Methanol*)	IZ: 25.0	Gentamicin (IZ: 32.0 MIC: 250)	[54]
			MIC: 900		
		*Ramalina sinensis* (*Ethanol*)	IZ: 23.0		
		*Ramalina sinensis* (*Acetone*)	IZ: 21.0		
		*Ramalina umeticola* (*Acetone*)	MIC: 31.25	Chloramphenicol (MIC: 7.81) Vancomycin (MIC: 7.81)	[53]
		*Ramalina hossei* (*Methanol*)	IZ: 13.3	Chloramphenicol (IZ: 34.0)	[55]
		*Ramalina conduplicans* (*Methanol*)	IZ: 15.0		
		*Ramalina pacifica* (*Methanol*)	IZ: 17.6		
			MIC: 1250	Streptomycin (MIC: 16)	[43]
		*Ramalina fraxinea* (*Acetone*)	MIC: 1250		
		*Ramalina farinacea* (*Acetone*)	MIC: 78		[36]
		*Cladonia foliacea* (*Chloroform*)	MIC: 0.48		[30]
		*Cladonia foliacea* (*Diethyl ether*)	MIC: 2.9		
		*Cladonia foliacea* (*Acetone*)	MIC: 7.8		
		*Cladonia foliacea* (*Ethanol*)	MIC: 3.9		
		*Cryptothecia striata* (*Methanol*)	IZ: 17.5		[31]
		*Cryptothecia striata* (*Ethanolic*)	IZ: 16.6		
		*Cryptothecia striata* (*Water*)	IZ: 14.0		
		*Cryptothecia scripta* (*Methanol*)	IZ: 22.0		
		*Cryptothecia scripta* (*Ethanolic*)	IZ: 15.0		
		*Cryptothecia scripta* (*Water*)	IZ: 12.5		
		*Phaeographis dendritica* (*Acetone*)	MIC: 125		[32]
		*Phaeographis dendriticaa* (*Methanol*)	MIC: 62.5		
		*Phaeographis dendritica* (*Benzene*)	MIC: 500		
		*Phaeographis dendritica* (*Diethyl ether*)	MIC: 250		
		*Trypethelevirens* (*Acetone*)	MIC: 250		
		*Trypethelevirens* (*Methanol*)	MIC: 125		
		*Trypethelevirens* (*Diethyl ether*)	MIC: 500		
		*Chloramphenicol* (*Acetone*)	MIC: 15.63	Chloramphenicol (MIC: 7.81) Vancomycin (MIC: 7.81)	[53]
	*Bacillus cereus*	*Ramalina hossei* (*Methanol*)	IZ: 22.0	Chloramphenicol (IZ: 36.6)	[55]
		*Ramalina conduplicans* (*Methanol*)			
		*Ramalina pacifica* (*Methanol*)	IZ: 27.0		
		*Ramalina fraxinea* (*Acetone*)	MIC: 1250	Streptomycin (MIC: 16)	[43]
		*Ramalina fastigiata* (*Acetone*)	MIC: 625		
		*Cladonia foliacea* (*Chloroform*)	MIC: 1.9		[30]
		*Cladonia foliacea* (*Diethyl ether*)	MIC: 46.8		
		*Cladonia foliacea* (*Acetone*)	MIC: 31.2		
		*Cladonia foliacea* (*Ethanol*)	MIC: 15.6		
	*Streptococcus pneumoniae*	*Usnea barbata* (*Acetone*)	IZ: 18.0	Ofloxacin (IZ: 19.0) Ceftriaxone (IZ: 32.3)	[49]
		*Usnea barbata* (*Ethanol*)	IZ: 18.3		
	*Staphylococcus epidermidis*	*Usnea steineri* (*Acetone*)	MIC < 10		[24]
	*Staphylococcus aureus*	*Usnea articulate* (*Methanol*)	IZ: 29.0/30.0	Gentamicin (IZ: 29.0)	[25]
		*Usnea antarctica* (*Methanol-acetone*)	IR: 94.76~100		[56]
		*Usnea aurantiaco-atraa* (*Methanol-acetone*)	IR: 98.43~100		
		*Usnea barbata* (*Methanol-acetone*)	MIC: 100		[21]
		*Usnea barbata* (*Acetone*)	IZ: 17.3	Ofloxacin (IZ: 26.3) Ceftriaxone (IZ: 25.0)	[49]
		*Usnea barbata* (*Ethanol*)	IZ: 12.3		
		*Usnea longissima* (*Methanol*)	IZ: 28.0	Streptomycin (IZ: 25.0)	[45]
		*Usnea longissima* (*Ethanol*)	IZ: 27.0		
		*Usnea longissima* (*Ethyl acetate*)	IZ: 25.0		
		*Usnea longissima* (*Acetone*)	IZ: 24.0		
		*Usnea blepharea* (*Acetone*)	IZ: 21.3	Amoxicillin (IZ: 22.0) Chloramphenicol (IZ: 30.8)	[48]
		*Usnea rubrotincta* (*Acetone*)	MIC: 125	Chloramphenicol (MIC: 31.25) Vancomycin (MIC: 15.63)	[53]
		*Usnea rubrotincta* (*Methanol*)	MIC: 500		
		*Usnea intermedia* (*Methanol*)	MIC: 128		[41]
		*Usnea filipendula* (*Methanol*)	MIC: 128		
		*Usnea fulvoreagens* (*Methanol*)	MIC: 512		
		*Parmelia conspersa* (*Methanol*)	MIC: 78.125	Amracin (MIC: 0.97)	[26]
		*Parmelia conspersa* (*Acetone*)	MIC: 312.5		
		*Parmelia perlata* (*Methanol*)	MIC: 156.25		
		*Parmelia perlata* (*Acetone*)	MIC: 312.5		
		*Parmelia caperata* (*Methanol*)	IZ: >19.0		[27]
		*Bulbothrix setschwanensis* (*Acetone*)	MIC: 1560	Rifampicin (MIC: 0.5)	[42]
		*Cetraria islandica* (*Acetone*)	RIZD: 92.44		[29]
		*Cetraria braunsiana* (*Methanol*)	IZ: 25.0	Streptomycin (IZ: 24.0)	[45]
		*Cetraria braunsiana* (*Ethanol*)	IZ: 24.0		
		*Cetraria braunsiana* (*Ethyl Acetate*)	IZ: 22.0		
		*Cetraria braunsiana* (*Acetone*)	IZ: 20.0		
		*Evernia prunastri* (*Dichloromethane*)	MIC: 4		[38]
		*Evernia prunastri* (*n-Hexane*)	MIC: 21		
		*Evernia prunastri* (*Acetonitrile*)	MIC: 14		
		*Evernia prunastri* (*Acetone*)	MIC: 78 RIZD: 62.9		[29,36]
		*Pseudever niafurfuracea* (*Acetone*)	MIC: 78 RIZD: 89.97		[36]
		*Pseudevernia furfuracea* (*Methanol*)	MIC: 1250	Gentamicin (MIC: 300)	[54]
		*Hypogymnia physodes* (*Methanol/Ethanol*)	MBC: 310		[33]
		*Ramalina sinensis* (*Methanol*)	IZ: 19.0 MIC > 7500	Gentamicin (IZ: 28.0 MIC: 300)	[54]
		*Ramalina sinensis* (*Ethanol*)	IZ: 16.0		
		*Ramalina sinensis* (*Acetone*)	IZ: 14.0		
		*Ramalina umeticola* (*Acetone*)	MIC: 31.25	Gentamicin (IZ: 28.0 MIC: 300) Chloramphenicol (MIC: 31.25) Vancomycin (MIC: 15.63)	[53]
		*Ramalina sinensis* (*Methanol*)	IZ: 26.0	Gentamicin (IZ: 29.0)	[25]
		*Ramalina fraxinea* (*Acetone*)	MIC: 20,000	Streptomycin (MIC: 31)	[43]
		*Ramalina fastigiata* (*Acetone*)	MIC: 10,000		
		*Ramalina farinacea* (*Acetone*)	MIC: 150		[36]
		*Cladonia incrassate* (*Acetone*)	MIC >40		[57]
		*Cladonia uncialis* (*Heptane*)	MIC: 5	Chloramphenicol (MIC: 5)	[58]
		*Cladonia uncialis* (*Diethyl ether*)	MIC: 2.5		
		*Cladonia uncialis* (*Acetone*)	MIC: 0.5		
		*Cladonia uncialis* (*Methanolic*)	MIC: 10		
		*Cladonia foliacea* (*Chloroform*)	MIC: 0.97		[30]
		*Cladonia foliacea* (*Diethyl ether*)	MIC: 0.73		
		*Cladonia foliacea* (*Acetone*)	MIC: 15.6		
		*Cladonia foliacea* (*Ethanol*)	MIC: 3.9		
		*Xanthoria plitti* (*Methanol*)	MIC: 7.8 IZ: 14.0	Gentamicin (IZ: 29.0)	[25]
		*Xanthoria parietina* (*Acetone*)	MIC: 15.6	Cefotaxime (MIC: 2) Benzyl Penicillin Sodium (MIC: 0.03) Tetracycline (MIC: 2)	[39]
		*Cryptothecia striata* (*Methanol*)	IZ: 16.5		[31]
		*Cryptothecia striata* (*Ethanolic*)	IZ: 16		
		*Cryptothecia striata* (*Water*)	IZ: 14		
		*Cryptothecia scripta* (*Methanol*)	IZ: 22		
		*Cryptothecia scripta* (*Ethanolic*)	IZ: 17		
		*Cryptothecia scripta* (*Water*)	IZ: 14		
		*Physcia parietina* (*Methanol*)	IZ: 9.0	Gentamicin (IZ: 29.0)	[25]
		*Heterodermia speciosa* (*Methanol*)	IZ: 7.0		[21]
		*Lobaria pulmonaria* (*Acetone*)	RIZD: 105.41		[29]
		*Stereocaulon tomentosum* (*Acetone*)	RIZD: 81.51		
		*Phaeographis dendritica* (*Acetone*)	MIC: 250		[32]
		*Phaeographis dendriticaa* (*Methanol*)	MIC: 125		
		*Phaeographis dendritica* (*Benzene*)	MIC: 250		
		*Phaeographis dendritica* (*Diethyl ether*)	MIC: 500		
		*Trypethelevirens* (*Acetone*)	MIC: 500		
		*Trypethelevirens* (*Methanol*)	MIC: 250		
		*Trypethelevirens* (*Benzene*)	MIC: 500		
		*Trypethelevirens* (*Diethyl ether*)	MIC: 500		
		*Chloramphenicol* (*Acetone*)	MIC: 31.25	Chloramphenicol (MIC: 31.25) Vancomycin (MIC: 15.63)	[53]
	*Staphylococcus aureus 33591*	*Usnea intermedia* (*Methanol*)	MIC ≥ 512		[41]
		*Usnea filipendula* (*Methanol*)	MIC: 256		
		*Usnea fulvoreagens* (*Methanol*)	MIC: 256		
	Methicillin-resistant *Staphylococcus aureus*	*Usnea* sp. *407* (*Acetone*)	IZ: 11.8	Vancomycin (MIC: 25) Cefotaxime (MIC > 256)	[28]
		*Usnea* cf. *scabrida 519* (*Acetone*)	IZ: 10.3		
		*Usnea* sp. *523* (*Acetone*)	IZ: 9.8		
		*Usnea* sp. *466* (*Acetone*)	IZ: 9.5		
		*Everniastrum* sp. *419* (*Acetone*)	IZ: 12.0		
		*Evernia mesomorpha 458* (*Acetone*)	IZ: 9.0		
		*Evernia prunastri* (*Acetone*)	MIC: 39		[36]
		*Pseudever niafurfuracea* (*Acetone*)	MIC: 39		
		*Parmotrema ramoddense* (*Ethanol*)	MIC: 96 IZ: 9.6	Vancomycin (IZ: 14.2)	[40]
		*Parmotrema tinctorum* (*Ethanol*)	MIC: 2400 IZ: 6.9		
		*Parmotrema tinctorum* (*Hexane*)	MIC: 2400 IZ: 7.1	Vancomycin (IZ: 12.7)	
		*Parmotrema ramoddense* (*Hexane*)	MIC: 60,000 IZ: 6.7	Vancomycin (IZ: 13.1)	
		*Parmotrema ramoddense* (*Aqueous*)	MIC: 12,000 IZ: 6.4	Vancomycin (IZ: 13.5)	
		*Ramalina* sp. *517* (*Acetone*)	IZ: 9.0		[28]
		*Ramalina implexa* (*n-Hexane/Dichloromethane*)	MIC: 500	Teicoplanin (MIC: 1)	[35]
		*Roccella phycopsis* (*n-Hexane/Dichloromethane*)	MIC: 1000		
		*Ramalina farinacea* (*Acetone*)	MIC: 150		[36]
	Methicillin-susceptible *Staphylococcus aureus*	*Parmotrema tinctorum* (*Ethanol*)	MIC: 2400	Vancomycin (IZ: 20.8)	[40]
		*Parmotrema tinctorum* (*Hexane*)	MIC: 2400		
		*Parmotrema ramoddense* (*Ethanol*)	MIC: 19.2	Vancomycin (IZ:13.4)	
		*Parmotrema ramoddense* (*Hexane*)	MIC: 12,000		
		*Parmotrema ramoddense* (*Aqueous*)	MIC: 2400		
		*Rhizoplaca chrysoleuca 431* (*Acetone*)	IZ: 11.8	Vancomycin (MIC: 25) Cefotaxime (MIC > 256)	[28]
		*Rhizoplaca chrysoleuca 449* (*Acetone*)	IZ: 10.0		
	*Enterococcus faecium*	*Usnea* sp. *407* (*Acetone*)	IZ: 23.0		[28]
		*Usnea* sp. *471* (*Acetone*)	IZ: 15.0		
		*Usnea* sp. *472* (*Acetone*)	IZ: 15.0		
		*Usnea* cf. *scabrida 519* (*Acetone*)	IZ: 14.5		
		*Usnea* sp. *523* (*Acetone*)	IZ: 13.5		
		*Usnea* sp. *466* (*Acetone*)	IZ: 16.0		
		*Usnea articulata 511* (*Acetone*)	IZ: 12.5		
		*Usnea steineri* (*Acetone*)	MIC: 32		
		*Allocetraria ambigua 435* (*Acetone*)	IZ: 9.8	Vancomycin (MIC: 25) Cefotaxime (MIC > 256)	[28]
		*Everniastrum* sp. *412* (*Acetone*)	IZ: 13.0		
		*Everniastrum* sp. *419* (*Acetone*)	IZ: 14.5		
		*Everniastrum nepalense 442* (*Acetone*)	IZ: 10.5		
		*Evernia mesomorpha 458* (*Acetone*)	IZ: 17.0		
		*Evernia divaricata 433* (*Acetone*)	IZ: 13.0		
		*Parmotrema* sp. *514* (*Acetone*)	IZ: 15.0		
		*Flavocetraria cucullata 443* (*Acetone*)	IZ: 12.5		
		*Ramalina* sp. *462* (*Acetone*)	IZ: 9.8		
		*Ramalina* sp. *470* (*Acetone*)	IZ: 15.0		
		*Ramalina* sp. *517* (*Acetone*)	IZ: 17.0		
		*Ramalina* sp. *518* (*Acetone*)	IZ: 16.3		
		*Ramalina* sp. *493* (*Acetone*)	IZ: 14.5		
		*Ramalina implexa* (*n-Hexane/Dichloromethane*)	MIC: 500	Teicoplanin (MIC ≤ 0.5)	[35]
		*Roccella phycopsis* (*n-Hexane/Dichloromethane*)	MIC: 1000		
		*Niebla ceruchoides 473* (*Acetone*)	IZ: 10.3	Vancomycin (MIC: 25) Cefotaxime (MIC > 256)	[28]
		*Cladonia* sp. *504* (*Acetone*)	IZ: 10.5		
		*Xanthoria parietina* (*Acetone*)	MIC: 15.6	Benzyl Penicillin Sodium (MIC: 8) Tetracycline (MIC: 2)	[39]
		*Heterodermia* sp. *535* (*Acetone*)	IZ: 11.5	Vancomycin (MIC: 25) Cefotaxime (MIC > 256)	[28]
		*Lobaria* sp. *403* (*Acetone*)	IZ: 9.0		
		*Rhizoplaca chrysoleuca 449* (*Acetone*)	IZ: 23.8		
		*Thamnolia vermicularis 445* (*Acetone*)	IZ: 11.0		
	*Enterococcus casseliflavus*	*Usnea barbata* (*Methanol/Ethyl acetate*)	IZ: 20.0~22.0	Levofloxacin (IZ: 25.0) Tetracycline (IZ: 26.0)	[22]
	*Listeria innocua*	*Evernia prunastri* (*Acetone*)	MIC: 625		[36]
		*Pseudever niafurfuracea* (*Acetone*)	MIC: 310		
		*Ramalina farinacea* (*Acetone*)	MIC: 310		
	*Listeria monocytogenes*	*Cladonia foliacea* (*Chloroform*)	MIC: 0.12		[30]
		*Cladonia foliacea* (*Diethyl ether*)	MIC: 0.73		
		*Cladonia foliacea* (*Acetone*)	MIC: 3.9		
		*Cladonia foliacea* (*Ethanol*)	MIC: 3.9		
	*Micrococcus luteus*	*Cryptothecia striata* (*Methanol*)	IZ: 20.0		[31]
		*Cryptothecia striata* (*Ethanolic*)	IZ: 15.6		
		*Cryptothecia striata* (*Water*)	IZ: 13.0		
		*Cryptothecia scripta* (*Methanol*)	IZ: 20.5		
		*Cryptothecia scripta* (*Ethanolic*)	IZ: 16.0		
		*Cryptothecia scripta* (*Water*)	IZ: 15.0		
	*Streptococcus mutans*	*Usnea longissima* (*Methanol*)	IZ: 14.0	Streptomycin (IZ: 15.0)	[45]
		*Usnea longissima* (*Ethanol*)	IZ: 15.0		
		*Usnea longissima* (*Ethyl acetate*)	IZ: 12.0		
		*Usnea longissima* (*Acetone*)	IZ: 12.0		
		*Cetraria braunsiana* (*Methanol*)	IZ: 20.0		
		*Cetraria braunsiana* (*Ethanol*)	IZ: 18.0		
		*Cetraria braunsiana* (*Ethyl Acetate*)	IZ: 16.0		
		*Cetraria braunsiana* (*Acetone*)	IZ: 15.0		
	*Streptococcus pyogenes*	*Bulbothrix setschwanensis* (*Acetone*)	MIC: 6250	Rifampicin (MIC: 62.5)	[42]
	*Streptococcus faecalis*	*Cladonia foliacea* (*Chloroform*)	MIC: 0.24		[30]
		*Cladonia foliacea* (*Diethyl ether*)	MIC: 0.73		
		*Cladonia foliacea* (*Acetone*)	MIC: 0.97		
		*Cladonia foliacea* (*Ethanol*)	MIC: 0.97		
	*Micrococcus lysodeikticus*	*Parmelia crinite* (*Methanol*)	IZ: 28.0 MIC: 940		[27]
	*Mycobacterium smegmatis*	*Usnea laevis* (*Acetone*)	MIC: 6.25	Rifampicin (MIC: 0.2)	[23]
	*Mycobacterium smegmatis* (MDR-40)	*Usnea laevis* (*Acetone*)	MIC: 0.41	Rifampicin (MIC: 100)	[23]
	*Mycobacterium smegmatis* (MDR-R)	*Usnea laevis* (*Acetone*)	MIC: 0.81	Rifampicin (MIC > 200)	[23]
	*Mycobacterium tuberculosis* H37Ra	*Usnea laevis* (*Acetone*)	MIC: 25	Rifampicin (MIC: 0.2)	[23]
	*Mycobacterium tuberculosisuberculosis*	*Usnea steineri* (*Acetone*)	MIC: 32	Isoniazid (MIC: 0.03)	[24]
	*Mycobacterium tuberculosis* (MDR-V791)	*Usnea laevis* (*Acetone*)	MIC: 1.63	Rifampicin (MIC > 200)	[23]
	*Mycobacterium tuberculosis* (MDR-A8)	*Usnea laevis* (*Acetone*)	MIC: 6.25	Rifampicin (MIC: 100)	[23]
	*Mycobacterium kansasii*	*Usnea steineri* (*Acetone*)	MIC: 62	Isoniazid (MIC: 0.05)	[24]
	*Mycobacterium avium*	*Usnea steineri* (*Acetone*)	MIC: 62	Isoniazid (MIC: 1.0)	[24]
Gram-negative bacteria	*Salmonella typhi*	*Usnea longissima* (*n-Hexane*)	IZ: 12.0	Ampicillin (IZ: 17.0)	[37]
		*Ramalina sinensis* (*Methanol*)	MIC: 10,000 IZ: 26.0	Chloramphenicol (IZ: 27.0)	[25]
		*Xanthoria plitti* (*Methanol*)	MIC: 9		
		*Xanthoria parietina* (*Acetone*)	MIC: 15.6	Cefotaxime (MIC: 0.5) Benzyl Penicillin Sodium (MIC: 4) Tetracycline (MIC: 1)	[39]
		*Physcia parietina* (*Methanol*)	MIC: 4000 IZ: 11.0	Chloramphenicol (IZ: 27.0)	[25]
	*Vibrio cholerae*	*Cryptothecia striata* (*Methanol*)	IZ: 17.6		[31]
		*Cryptothecia striata* (*Ethanolic*)	IZ: 16.3		
		*Cryptothecia striata* (*Water*)	IZ: 12.0		
		*Cryptothecia scripta* (*Methanol*)	IZ: 19.0		
		*Cryptothecia scripta* (*Ethanolic*)	IZ: 18.3		
		*Cryptothecia scripta* (*Water*)	IZ: 13.5		
		*Phaeographis dendritica* (*Acetone*)	MIC: 62.5		[32]
		*Phaeographis dendriticaa* (*Methanol*)	MIC: 125		
		*Phaeographis dendritica* (*Benzene*)	MIC: 500		
		*Phaeographis dendritica* (*Diethyl ether*)	MIC: 250		
		*Trypethelevirens* (*Acetone*)	MIC: 125		
		*Trypethelevirens* (*Methanol*)	MIC: 62.5		
		*Trypethelevirens* (*Benzene*)	MIC: 250		
	*Aeromonas hydrophila*	*Cladonia foliacea* (*Chloroform*)	MIC: 3.9		
		*Cladonia foliacea* (*Diethyl ether*)	MIC: 46.8		
		*Cladonia foliacea* (*Acetone*)	MIC: 3.9		
		*Cladonia foliacea* (*Ethanol*)	MIC: 3.9		
	*Pseudomonas aeruginosa*	*Usnea articulate* (*Methanol*)	IZ: 28.0	Gentamicin (IZ: 26.0)	[25]
		*Usnea florida* (*Methanol*)	IZ: 18.0		
		*Usnea barbata* (*Methanol/Ethyl acetate*)	IZ: 16.0~20.0	Levofloxacin (IZ: 21.0) Tetracycline (IZ: 24.0)	[22]
		*Usnea barbata* (*Acetone*)	IZ: 17.0	Ofloxacin (IZ: 19.3) Ceftriaxone (IZ: 21.0)	[49]
		*Usnea barbata* (*Ethanol*)	IZ: 20.0		
		*Usnea longissima* (*Methanol*)	IZ: 16.0	Streptomycin (IZ: 15.0)	[45]
		*Usnea longissima* (*Ethanol*)	IZ: 15.0		
		*Usnea longissima* (*Ethyl acetate*)	IZ: 14.0		
		*Evernia prunastri* (*Dichloromethane*)	MIC: 167		[38]
		*Evernia prunastri* (*n-Hexane*)	MIC: 150		
		*Evernia prunastri* (*Acetonitrile*)	MIC: 133		
		*Ramalina hossei* (*Methanol*)	IZ: 13.0	Chloramphenicol (IZ: 29.0)	[55]
		*Ramalina conduplicans* (*Methanol*)	IZ: 20.0		
		*Ramalina pacifica* (*Methanol*)	IZ: 21.6		
		*Xanthoria parietina* (*Acetone*)	MIC: 15.6	Cefotaxime (MIC: 16) Tetracycline (MIC: 32)	[33]
		*Cryptothecia striata* (*Methanol*)	IZ: 17.0		[31]
		*Cryptothecia striata* (*Ethanolic*)	IZ: 14.3		
		*Cryptothecia striata* (*Water*)	IZ: 16.0		
		*Cryptothecia scripta* (*Methanol*)	IZ: 18.6		
		*Cryptothecia scripta* (*Ethanolic*)	IZ: 16.0		
		*Cryptothecia scripta* (*Water*)	IZ: 13.0		
	*Pseudomonas fluorescens*	*Usnea barbata* (*Methanol-acetone*)	IZ: 29.0		[52]
	*Enterobacter cloacae*	*Usnea florida* (*Methanol*)	IZ: 25.0	Gentamicin (IZ: 27.0) Tetracycline (IZ: 16.0)	[25]
		*Ramalina sinensis* (*Methanol*)	IZ: 17.0		
		*Xanthoria parietina* (*Acetone*)	MIC: 15.6	Benzyl Penicillin Sodium (MIC: 4)	[39]
	*Enterobacter cloacae* CI	*Xanthoria parietina* (*Acetone*)	MIC: 62.5		
	*Enterobacter aerogenes*	*Xanthoria parietina* (*Acetone*)	MIC: 15.6	Benzyl Penicillin Sodium (MIC: 4)	[39]
	*Enterobacter aerogenes* CI	*Xanthoria parietina* (*Acetone*)	MIC: 62.5		
	*Escherichia coli*	*Usnea florida* (*Methanol*)	MIC: 8000 IZ: 27.0	Gentamicin (IZ: 22.0)	[25]
		*Usnea longissima* (*Methanol*)	IZ: 34.0	Streptomycin (IZ: 28.0)	[45]
		*Usnea longissima* (*Ethanol*)	IZ: 32.0		
		*Usnea longissima* (*Ethyl acetate*)	IZ: 28.0		
		*Usnea longissima* (*Acetone*)	IZ: 26.0		
		*Usnea longissima* (*n-Hexane*)	IZ: 14.0	Ampicillin (IZ: 21.0)	[37]
		*Usnea longissima* (*n-Hexane*)	IZ: 17.0	Amoxicillin (IZ: 15.8) Chloramphenicol (IZ: 31.2)	[48]
		*Parmelia conspersa* (*Methanol*)	MIC: 39.1	Amracin (MIC: 0.97)	[26]
		*Parmelia conspersa* (*Acetone*)	MIC: 78.125		
		*Parmelia perlata* (*Methanol*)	MIC: 39.1		
		*Parmelia perlata* (*Acetone*)	MIC: 39.1		
		*Parmelia crinite* (*Methanol*)	IZ: 15.0 MIC: 3750		[27]
		*Parmelia sulcata* (*Acetone*)	IZ: 24.0 MIC: 1560		
	
		*Bulbothrix setschwanensis* (*Acetone*)	MIC: 6250	Rifampicin (MIC: 4)	[42]
		*Cetraria braunsiana* (*Methanol*)	IZ: 22.0	Streptomycin (IZ: 22.0)	[45]
		*Cetraria braunsiana* (*Ethanol*)	IZ: 20.0		
		*Cetraria braunsiana* (*Ethyl Acetate*)	IZ: 20.0		
		*Cetraria braunsiana* (*Acetone*)	IZ: 18.0		
		*Evernia prunastri* (*Dichloromethane*)	MIC: 500		[38]
		*Evernia prunastri* (*n-Hexane*)	MIC > 500		
		*Evernia prunastri* (*Acetonitrile*)	MIC: 250		
		*Ramalina sinensis* (*Methanol*)	IZ: 18.0	Gentamicin (IZ: 22.0)	[25]
		*Ramalina hossei* (*Methanol*)	IZ: 13.0	Chloramphenicol (IZ: 26.6)	[55]
		*Ramalina conduplicans* (*Methanol*)	IZ: 14.3		
		*Ramalina pacifica* (*Methanol*)	IZ: 16.0		
		*Ramalina fastigiata* (*Acetone*)	MIC: 20,000	Streptomycin (MIC: 62)	[43]
		*Ramalina implexa* (*n-Hexane/Dichloromethane*)	MIC: 500	Colistin (MIC: 1)	[35]
		*Roccella phycopsis* (*n-Hexane/Dichloromethane*)	MIC: 1000		
		*Cladonia uncialis* (*Heptane*)	MIC: 1000	Chloramphenicol (MIC: 100)	[58]
		*Cladonia uncialis* (*Diethyl ether*)	MIC: 1000		
		*Cladonia uncialis* (*Acetone*)	MIC: 100		
		*Cladonia uncialis* (*Methanolic*)	MIC: 1000		
		*Xanthoria plitti* (*Methanol*)	MIC: 10 IZ: 12.0	Gentamicin (IZ: 22.0)	[25]
		*Cryptothecia striata* (*Methanol*)	IZ: 17.3		[31]
		*Cryptothecia striata* (*Ethanolic*)	IZ: 16.0		
		*Cryptothecia striata* (*Water*)	IZ: 12.0		
		*Cryptothecia scripta* (*Methanol*)	IZ: 23.0		
		*Cryptothecia scripta* (*Ethanolic*)	IZ: 16.6		
		*Cryptothecia scripta* (*Water*)	IZ: 13.0		
		*Physcia parietina* (*Methanol*)	MIC: 4000 IZ: 10.0	Gentamicin (IZ: 22.0)	[25]
		*Phaeographis dendritica* (*Acetone*)	MIC: 125		[32]
		*Phaeographis dendriticaa* (*Methanol*)	MIC: 125		
		*Phaeographis dendritica* (*Benzene*)	MIC: 120~125		
		*Phaeographis dendritica* (*Diethyl ether*)	MIC: 500		
		*Trypethelevirens* (*Acetone*)	MIC: 250		
		*Trypethelevirens* (*Methanol*)	MIC: 500		
		*Trypethelevirens* (*Benzene*)	MIC: 250		
		*Trypethelevirens* (*Diethyl ether*)	MIC: 500		
	*Escherichia coli (E245,O157:H7)*	*Usnea intermedia* (*Methanol*)	MIC: 64		[41]
		*Usnea filipendula* (*Methanol*)	MIC: 64		
		*Usnea fulvoreagens* (*Methanol*)	MIC: 64		
	*Escherichia coli (E103,121,224,246,248,300,25922)*	*Usnea intermedia* (*Methanol*)	MIC: 128		[41]
		*Usnea filipendula* (*Methanol*)	MIC: 128		
		*Usnea fulvoreagens* (*Methanol*)	MIC: 128		
	*Escherichia coli 101*	*Usnea intermedia* (*Methanol*)	MIC: 256		[41]
		*Usnea filipendula* (*Methanol*)	MIC: 512		
		*Usnea fulvoreagens* (*Methanol*)	MIC ≥ 512		
	*Escherichia coli (25922,O157:H7)*	*Usnea fulvoreagens* (*Methanol*)	MIC: 512		[41]
	*Proteus mirabilis*	*Usnea articulate* (*Methanol*)	MIC: 9000 IZ: 21.0	Gentamicin (IZ: 22.0)	[25]
		*Parmelia conspersa* (*Methanol*)	MIC: 39.1	Amracin (MIC: 0.49)	[26]
		*Parmelia conspersa* (*Acetone*)	MIC: 78.125		
		*Parmelia perlata* (*Methanol*)	MIC: 156.25		
		*Parmelia perlata* (*Acetone*)	MIC: 78.125		
		*Pseudevernia furfuracea* (*Methanol*)	MIC: 630		[33]
		*Ramalina sinensis* (*Methanol*)	IZ: 18.0	Gentamicin (IZ: 22.0)	[25]
		*Ramalina fraxinea* (*Acetone*)	MIC: 10,000	Streptomycin (MIC: 62)	[43]
		*Ramalina fastigiata* (*Acetone*)	MIC: 5000	Streptomycin (MIC: 62)	
		*Xanthoria parietina* (*Acetone*)	MIC: 15.6	Cefotaxime (MIC: 0.03) Benzyl Penicillin Sodium (MIC: 4) Tetracycline (MIC: 32)	[39]
		*Xanthoria plitti* (*Methanol*)	IZ: 11.0	Gentamicin (IZ: 22.0)	[25]
		*Physcia parietina* (*Methanol*)	IZ: 10.0		
	*Proteus mirabilis* CI	*Xanthoria parietina* (*Acetone*)	MIC: 15.6	Cefotaxime (MIC: 32)	[39]
	*Proteus rettgeri*	*Usnea articulate* (*Methanol*)	IZ: 23.0	Gentamicin (IZ: 21.0)	[25]
		*Usnea florida* (*Methanol*)	IZ: 23.0		
		*Ramalina sinensis* (*Methanol*)	IZ: 22.0		
		*Xanthoria plitti* (*Methanol*)	MIC: 7 IZ: 10.0		
	*Proteus vulgaris*	*Usnea articulate* (*Methanol*)	IZ: 29.0	Gentamicin (IZ: 24.0) Tetracycline (IZ: 10.0)	[25]
		*Usnea florida* (*Methanol*)	IZ: 29.0		
		*Parmelia conspersa* (*Methanol*)	MIC: 39.1	Amracin (MIC: 0.49)	[26]
		*Parmelia conspersa* (*Acetone*)	MIC: 78.125		
		*Parmelia perlata* (*Methanol*)	MIC: 78.125		
		*Parmelia perlata* (*Acetone*)	MIC: 78.125		
		*Ramalina sinensis* (*Methanol*)	IZ: 25.0	Gentamicin (IZ: 24.0) Tetracycline (IZ: 10.0)	[25]
		*Cladonia foliacea* (*Chloroform*)	MIC: 3.9		[30]
		*Cladonia foliacea* (*Diethyl ether*)	MIC: 46.8		
		*Cladonia foliacea* (*Acetone*)	MIC: 3.9		
		*Cladonia foliacea* (*Ethanol*)	MIC: 3.9		
		*Xanthoria parietina* (*Acetone*)	MIC: 15.6	Cefotaxime (MIC: 2) Benzyl Penicillin Sodium (MIC: 4)	[39]
	*Proteus vulgaris* CI	*Xanthoria parietina* (*Acetone*)	MIC: 15.6	Cefotaxime (MIC: 32)	[39]
	*Citrobacter youngae*	*Usnea articulate* (*Methanol*)	MIC: 4000 IZ: 16.0	Gentamicin (IZ: 24.0) Tetracycline (IZ: 10.0)	[25]
		*Usnea florida* (*Methanol*)	MIC: 6000 IZ: 16.0		
		*Ramalina sinensis* (*Methanol*)	IZ: 24.0		
	*Citrobacter freundii*	*Usnea florida* (*Methanol*)	MIC: 5000 IZ: 19.0	Gentamicin (IZ: 23.0) Tetracycline (IZ: 15.0)	[25]
		*Ramalina sinensis* (*Methanol*)	IZ: 21.0		
		*Xanthoria plitti* (*Methanol*)	IZ: 12.0		
		*Physcia parietina* (*Methanol*)	IZ: 11.0		
	*Salmonella enterica*	*Usnea articulate* (*Methanol*)	MIC: 8000	Gentamicin (IZ: 18.0)	[25]
		*Usnea florida* (*Methanol*)	MIC: 10,000		
	*Agrobacterium tumefaciens*	*Usnea longissima* (*Methanol*)	IZ: 24.0	Streptomycin (IZ: 18.0)	[45]
		*Usnea longissima* (*Ethanol*)	IZ: 22.0		
		*Usnea longissima* (*Ethyl acetate*)	IZ: 23.0		
		*Usnea longissima* (*Acetone*)	IZ: 21.0		
		*Cetraria braunsiana* (*Methanol*)	IZ: 20.0		
		*Cetraria braunsiana* (*Ethanol*)	IZ: 25.0		
		*Cetraria braunsiana* (*Ethyl Acetate*)	IZ: 18.0		
		*Cetraria braunsiana* (*Acetone*)	IZ: 16.0		
	*Klebsiella pneumoniae*	*Parmelia conspersa* (*Methanol*)	MIC: 156.25	Amracin (MIC: 0.49)	[26]
		*Parmelia conspersa* (*Acetone*)	MIC156.25		
		*Parmelia perlata* (*Methanol*)	MIC: 156.25		
		*Parmelia perlata* (*Acetone*)	MIC: 156.25		
		*Roccella phycopsis* (*n-Hexane/Dichloromethane*)	MIC: 1000	Colistin (MIC < 2)	[35]
		*Xanthoria parietina* (*Acetone*)	MIC: 62.5	Cefotaxime (MIC: 1) Tetracycline (MIC: 16)	[39]
	*Providencia rettgeri*	*Ramalina sinensis* (*Methanol*)	MIC: 8000	Gentamicin (IZ: 21.0)	[25]
		*Physcia parietina* (*Methanol*)	MIC: 8000 IZ: 11.0		
	*Acinetobacter baumannii*	*Ramalina implexa* (*n-Hexane/Dichloromethane*)	MIC: 500/1000	Colistin (MIC: 0.78)	[35]
	*Shigella flexneri*	*Cryptothecia striata* (*Methanol*)	IZ: 18.3		[31]
		*Cryptothecia striata* (*Ethanolic*)	IZ: 15.6		
		*Cryptothecia striata* (*Water*)	IZ: 14.5		
		*Cryptothecia scripta* (*Methanol*)	IZ: 20.0		
		*Cryptothecia scripta* (*Ethanolic*)	IZ: 19.0		
		*Cryptothecia scripta* (*Water*)	IZ: 12.0		
	*Shigella dysenteriae*	*Cryptothecia striata* (*Methanol*)	IZ: 18.5		[31]
		*Cryptothecia striata* (*Ethanolic*)	IZ: 16.0		
		*Cryptothecia striata* (*Water*)	IZ: 15.0		
		*Cryptothecia scripta* (*Methanol*)	IZ: 21.5		
		*Cryptothecia scripta* (*Water*)	IZ: 13.0		
Fungi	*Cryptococcus neoformans*	*Bulbothrix setschwanensis* (*Acetone*)	MIC: 6250	Amphotericin B (MIC: 1.44)	[42]
	*Candida albicans*	*Usnea barbata* (*Methanol/Ethyl acetate*)	IZ: 13.0~16.0	Fluconazole (IZ: 32.3) Voriconazole (IZ: 34.3)	[22]
		*Usnea longissima* (*Methanol*)	IZ: 15.0	Ketoconazole (IZ: 10.0)	[45]
		*Usnea longissima* (*Ethanol*)	IZ: 16.0		
		*Usnea longissima* (*Ethanol*)	IZ: 11.0		[44]
		*Usnea longissima* (*Ethyl acetate*)	IZ: 12.0	Ketoconazole (IZ: 10.0)	[45]
		*Usnea longissima* (*Acetone*)	IZ: 14.0		
		*Parmelia conspersa* (*Methanol*)	MIC: 39.1	Ketoconazole (MIC: 1.95)	[26]
		*Parmelia conspersa* (*Acetone*)	MIC: 39.1		
		*Parmelia perlata* (*Methanol*)	MIC: 78.125		
		*Parmelia perlata* (*Acetone*)	MIC: 78.125		
		*Parmelia perlata* (*Methanol*)	MIC: 78.125	Ketoconazole (MIC: 1.95)	[26]
		*Parmelia sulcata* (*Acetone*)	MIC: 780		[27]
		*Cetraria braunsiana* (*Methanol*)	IZ: 25.0	Ketoconazole (IZ: 14.0)	[45]
		*Cetraria braunsiana* (*Ethanol*)	IZ: 30.0		
		*Cetraria braunsiana* (*Ethyl Acetate*)	IZ: 28.0		
		*Cetraria braunsiana* (*Acetone*)	IZ: 27.0		
		*Evernia prunastri* (*Dichloromethane*)	MIC: 150		[38]
		*Evernia prunastri* (*n-Hexane*)	MIC: 150		
		*Evernia prunastri* (*Acetonitrile*)	MIC: 38/IZ: 12 MIC > 7.5		
		*Ramalina fraxinea* (*Acetone*)	MIC: 5000	Ketoconazole (MIC: 39)	[43]
		*Ramalina fastigiata* (*Acetone*)	MIC: 625		
		*Cladonia uncialis* (*Heptane*)	MIC: 750	Amphothericin B (MIC: 1)	[58]
		*Cladonia uncialis* (*Diethyl ether*)	MIC: 750		
		*Cladonia uncialis* (*Acetone*)	MIC: 750		
		*Cladonia uncialis* (*Methanolic*)	MIC: 250		
		*Cladonia foliacea* (*Chloroform*)	MIC: 500		[30]
		*Cladonia foliacea* (*Diethyl ether*)	MIC: 375		
		*Cladonia foliacea* (*Acetone*)	MIC: 500		
		*Cladonia foliacea* (*Ethanol*)	MIC: 500		
		*Xanthoria plitti* (*Methanol*)	MIC: 7 IZ: 10.0	Gentamicin (IZ: 21.0)	[25]
		*Heterodermia diademata* (*Ethyl acetate*)	MIC: 230		[21]
		*Phaeographis dendritica* (*Acetone*)	MIC: 250		[32]
		*Phaeographis dendriticaa* (*Methanol*)	MIC: 125		
		*Phaeographis dendritica* (*Benzene*)	MIC: 120~125		
		*Phaeographis dendritica* (*Diethyl ether*)	MIC: 500		
		*Trypethelevirens* (*Acetone*)	MIC: 250		
		*Trypethelevirens* (*Methanol*)	MIC: 500		
		*Trypethelevirens* (*Benzene*)	MIC: 250		
		*Trypethelevirens* (*Diethyl ether*)	MIC: 250		
	*Candida albicans* CI	*Xanthoria parietina* (*Acetone*)	MIC > 100	Ketoconazole (MIC: 0.4)	[39]
	*Candida parapsilosis*	*Usnea barbata* (*Ethyl acetate*)	IZ: 7.0	Fluconazole (IZ: 25.7) Voriconazole (IZ: 30.7)	[22]
	*Candida glabrata*	*Cladonia foliacea* (*Chloroform*)	MIC: 500		[30]
		*Cladonia foliacea* (*Diethyl ether*)	MIC: 375		
		*Cladonia foliacea* (*Acetone*)	MIC: 500		
		*Cladonia foliacea* (*Ethanol*)	MIC: 500		
	*Cladosporium cladosporioides*	*Ramalina fraxinea* (*Acetone*)	MIC: 5000	Ketoconazole (MIC: 39)	[43]
		*Ramalina fastigiata* (*Acetone*)	MIC: 2500		
	*Fusarium oxysporum*	*Usnea longissima* (*Methanol*)	IZ: 14.0	Ketoconazole (IZ: 12.0)	[45]
		*Usnea longissima* (*Ethanol*)	IZ: 12.0		
		*Usnea longissima* (*Ethyl acetate*)	IZ: 12.0		
		*Usnea longissima* (*Acetone*)	IZ: 10.0		
		*Usnea hirta* (*Methanol*)	IZ: 11.3 MIC: 3.125		[44]
		*Usnea hirta* (*Acetone*)	IZ: 12.6 MIC: 6.25		
		*Cetraria braunsiana* (*Methanol*)	IZ: 22.0	Ketoconazole (IZ: 12.0)	[45]
		*Cetraria braunsiana* (*Ethanol*)	IZ: 25.0		
		*Cetraria braunsiana* (*Ethyl Acetate*)	IZ: 24.0		
		*Cetraria braunsiana* (*Acetone*)	IZ: 22.0		
		*Ramalina fraxinea* (*Acetone*)	MIC: 5000	Ketoconazole (MIC: 78)	[43]
		*Ramalina fastigiata* (*Acetone*)	MIC: 2500		
	*Fusarium fujikuroi*	*Bryoria capillaris* (*Acetone*)	MIC: 156.2	Amphotericin B (MIC: 3) Isavuconazole (MIC: 5) Natamycin (MIC: 4) Posaconazole (MIC: 0.65) Voriconazole (MIC: 3.7) Fluconazole (MIC: 90) Itraconazole (MIC: 27)	[47]
		*Bryoria capillaris* (*Methanol*)	MIC: 312.5		
		*Parmotrema andinum* (*Propyl alcohol*)	IZ: 20.7		
		*Gyalolechia subbracteata* (*Methyl alcohol*)	IZ: 33.3		
		*Pyrenodesmia variabilis* (*Methyl alcohol*)	IZ: 27.3		
		*Blennothallia crispa* (*Methyl alcohol*)	IZ: 28.0		
		*Catapyrenium squamulosum* (*Acetone*)	IZ: 5.0		
	*Fusarium solani*	*Alectoria sarmentosa* (*Ethanol*)	IZ: 25.0	AmphotericinB (MIC: 10) Flucytosine (MIC: 410) Itraconazole (MIC: 37) Voriconazole (MIC: 12)	[47]
		*Bryoria capillaris* (*Acetone*)	MIC: 156.2		
		*Bryoria capillaris* (*Methanol*)	MIC: 312.5		
		*Parmotrema andinum* (*Propyl alcohol*)	IZ: 19.0		
		*Parmotrema austrosinense* (*Ethyl acetate*)	IZ: 12.3		
		*Parmotrema grayanum* (*Ethyl acetate*)	IZ: 15.3		
		*Parmotrema grayanum* (*Acetone*)	IZ: 17 IR: 89		
		*Parmotrema thomsonii* (*Trichloromethane*)	IZ: 18.0		
		*Parmotrema tinctorum* (*Ethyl acetate*)	IZ: 18.6		
		*Flavoparmelia caperata* (*Acetone/Chloroform*)	IZ: 10.3		
		*Hypogymnia nepalensis* (*Acetone*)	IZ: 16.0		
		*Roccella montagnei* (*Methanol/Ethyl acetate*)	IZ: 13.3		
		*Cladonia rangiferina* (*Ethanol*)	IZ: 16.0		
		*Heterodermia diademata* (*Chloroform*)	IZ: 20.0		
		*Teloschistes flavicans* (*Acetone*)	IZ: 18.6		
	*Fusarium* sp.	*Ramalina hossei* (*Methanol*)	IZ: 22.0	Self-comparison (IZ: 34.6)	[55]
		*Ramalina conduplicans* (*Methanol*)	IZ: 22.0		
		*Ramalina pacifica* (*Methanol*)	IZ: 23.0		
	*Fusarium udum Butler*	*Parmelia reticulate* (*n-Hexane*)	ED_50_: 43.7		[27]
	*Schizophyllum commune*	*Usnea barbata* (*Methanol-acetone*)	IR: 51.60		[21]
	*Alternaria alternata*	*Usnea barbata* (*Methanol-acetone*)	IR: 100		[21]
		*Ramalina fraxinea* (*Acetone*)	MIC: 5000	Ketoconazole (MIC: 78)	[43]
		*Ramalina fastigiata* (*Acetone*)	MIC: 2500		
	*Trichoderma viride*	*Usnea longissima* (*Ethanol*)	IZ: 14.0		[44]
		*Ramalina fraxinea* (*Acetone*)	MIC: 5000	Ketoconazole (MIC: 78)	[43]
		*Ramalina fastigiata* (*Acetone*)	MIC: 2500		
	*Aspergillus niger*	*Parmelia conspersa* (*Methanol*)	MIC: 39.1	Ketoconazole (MIC: 0.97)	[26]
		*Parmelia conspersa* (*Acetone*)	MIC: 39.1		
		*Parmelia perlata* (*Methanol*)	MIC: 39.1		
		*Parmelia perlata* (*Acetone*)	MIC: 19.53		
		*Cetraria braunsiana* (*Methanol*)	IZ: 14.0	Ketoconazole (IZ: 12.0)	[45]
		*Cetraria braunsiana* (*Ethanol*)	IZ: 14.0		
		*Cetraria braunsiana* (*Ethyl Acetate*)	IZ: 12.0		
		*Cetraria braunsiana* (*Acetone*)	IZ: 12.0		
		*Ramalina fraxinea* (*Acetone*)	MIC: 10,000	Ketoconazole (MIC: 78)	[43]
		*Ramalina fastigiata* (*Acetone*)	MIC: 10,000		
		*Phaeographis dendritica* (*Acetone*)	MIC: 500		[32]
		*Phaeographis dendriticaa* (*Methanol*)	MIC: 250		
		*Phaeographis dendritica* (*Benzene*)	MIC: 500		
		*Phaeographis dendritica* (*Diethyl ether*)	MIC: 250		
		*Trypethelevirens* (*Acetone*)	MIC: 500		
		*Trypethelevirens* (*Methanol*)	MIC: 250		
		*Trypethelevirens* (*Diethyl ether*)	MIC: 500		
	*Aspergillus flavus* *Mucor mucedo*	*Ramalina fastigiata* (*Acetone*)	MIC: 20,000		[43]
		*Parmelia sulcata* (*Acetone*)	MIC: 780		[27]
		*Ramalina fraxinea* (*Acetone*)	MIC: 10,000		
		*Ramalina fastigiata* (*Acetone*)	MIC: 5000	Ketoconazole (MIC: 156)	[43]
	*Saccharomyces cerevisiae*	*Parmelia sulcata* (*Acetone*)	MIC: 780		[27]
	*Rhizoctonia bataticola*	*Parmelia reticulate* (*n-Hexane*)	ED_50_: 25.1		[27]
	*Rhizoctonia solani Kühn*	*Parmelia reticulate* (*n-Hexane*)	ED_50_: 29.4		[27]
		*Xanthoria parietina* (*Acetone*)	MIC: 62.5	Ketoconazole (MIC: 0.2)	[39]
	*Sclerotium rolfsii Sacc*	*Parmelia reticulate* (*n-Hexane*)	ED_50_: 43.7		[27]
	*Alternaria* sp.	*Ramalina hossei* (*Methanol*)	IZ: 6.6	Self-comparison (IZ: 51.0)	[55]
		*Ramalina conduplicans* (*Methanol*)	IZ: 13.0		
		*Ramalina pacifica* (*Methanol*	IZ: 18.0		
	*Curvularia* sp.	*Ramalina hossei* (*Methanol*)	IZ: 18.0	Self-comparison (IZ: 47.0)	[55]
		*Ramalina conduplicans* (*Methanol*)	IZ: 21.3		
		*Ramalina pacifica* (*Methanol*)	IZ: 22.0		
	*Penicillium chrysogenum*	*Ramalina fraxinea* (*Acetone*)	MIC: 10,000	Ketoconazole (MIC: 78)	[43]
	*Penicillium expansum*	*Ramalina fraxinea* (*Acetone*)	MIC: 20,000	Ketoconazole (MIC: 156)	[43]
		*Ramalina fastigiata* (*Acetone*)	MIC: 20,000		
	*Penicillium chrysogenum*	*Ramalina fastigiata* (*Acetone*)	MIC: 5000	Ketoconazole (MIC: 78)	[43]
	*Penicillium verrucosum*	*Phaeographis dendritica* (*Acetone*)	MIC: 125		[32]
		*Phaeographis dendriticaa* (*Methanol*)	MIC: 62.5		
		*Phaeographis dendritica* (*Diethyl ether*)	MIC: 500		
		*Trypethelevirens* (*Diethyl ether*)	MIC: 500		
		*Trypethelevirens* (*Acetone*)	MIC: 125		
		*Trypethelevirens* (*Benzene*)	MIC: 250		
	*Botrytis cinerea*	*Xanthoria parietina* (*Acetone*)	MIC > 100	Ketoconazole (MIC: 0.2)	[39]
		*Phaeographis dendritica* (*Acetone*)	MIC: 125		[32]
		*Phaeographis dendriticaa* (*Methanol*)	MIC: 125		
		*Trypethelevirens* (*Diethyl ether*)	MIC: 500		
		*Phaeographis dendritica* (*Diethyl ether*)	MIC: 125		
		*Trypethelevirens* (*Acetone*)	MIC: 500		
	*Achlya bisexualis*	*Usnea longissima* (*Acetone*)	MIC: 200		[46]
		*Cladonia amaurocraea* (*Acetone*)	MIC: 200		
		*Cladonia rangiferina* (*Acetone*)	MIC: 200		
	*Saprolegnia parasitica*	*Usnea longissima* (*Acetone*)	MIC: 200		[46]
		*Cladonia amaurocraea* (*Acetone*)	MIC: 200		
		*Cladonia rangiferina* (*Acetone*)	MIC: 200		
	*Pythium debaryanum*	*Parmelia reticulate* (*Ethyl acetate*)	ED_50_: 48.4		[27]
	*Pythium* sp.	*Usnea longissima* (*Acetone*)	MIC: 800		[45]
		*Cladonia amaurocraea* (*Acetone*)	MIC: 800		[46]
		*Cladonia rangiferina* (*Acetone*)	MIC: 1600		

CI: clinical isolate strain; MIC: minimum inhibitory concentration; MBC: minimum bactericidal concentration; ED_50_: effective dose 50; IZ: inhibition zone diameter; IR: inhibition rate; RIZD (%): relative inhibition zone diameter.

## 3. Antimicrobial Active Compounds of Lichens

Lichens secondary metabolites are favored by researchers for their rich diversity of chemical structures [56]. These metabolites, which primarily originate from the secondary metabolic pathway of lichens, include fatty (aliphatic) and phenolic compounds, which are usually deposited on the surface of the mycelial cells in the form of water-insoluble crystals [59]. Among the more than 800 lichen chemicals currently identified, up to 82% are lichen-specific [41]. Chemical taxonomy studies revealed that lichen-specific secondary metabolites mainly include depsides, depsidones, and dibenzofuran derivatives [23]. Among them, lichen chemicals with antimicrobial activity are mainly synthesized through the acetate–polymalonate pathway, including depsides (carboxylic acid derivatives) and its derivatives, usnic acid and related products, anthraquinones, and higher fatty acids and esters. While terpenoids are mainly synthesized through the mevalonic acid pathway, pulvinic acid derivatives are mainly derived from the shikimic acid pathway [59], and the discovery of these substances provides a solid scientific foundation for the study of the bioactivity of lichens and their potential applications.

### 3.1. Phenol (Carboxylic Acid) Derivatives

As central antimicrobial active chemical constituents of lichens, phenolic compounds distinguish themselves with their unique chemical structures, biological activities, ecological distribution, environmental adaptations, chemical defenses, biosynthetic complexities, structure–activity relationships, rarity, and distinctiveness [60]. These compounds contribute to the lichens’ adaptation to extreme environments and also play a role in defending against pathogens and herbivores [61]. Phenolic acid compounds in lichens consist of monocyclic derivatives, depsides, depsidones, dibenzofuran derivatives, and a small amount of anthraquinones and xanthones [62]. Their diversity and complexity offer significant potential for applications in medicine, agriculture, and industry, particularly in the exploration of novel anti-infective strategies and biopesticides.

#### 3.1.1. Monocyclic Derivatives

Lichen monocyclic derivatives, by introducing diverse functional groups such as methoxy, hydroxyl, aldehyde, carboxyl, ester bonds, and halogens, form a family of compounds rich in biological activities [63]. For instance, 4-chlororcinol (MIC: 1–17 µg/mL) and orcinol (MIC: 18.75 µg/mL) exhibited potent activity against methicillin-resistant *Staphylococcus aureus* [35], methyl β-orcinol-carboxylate inhibited *Streptococcus gordonii* (MIC: 375 µg/mL) [64], and orsellinic acid combated *Fusarium fujikuroi* (MIC: 15.1 µg/mL) [47]. Methyl *β*-orsellinate inhibited *Staphylococcus aureus* and *Helicobacter pylori*, and with a notable effect against *Helicobacter pylori* (IZ:27 mm) [44]. Compounds such as ethyl everninate, dibutyl phthalate, and methyl-2,4-dihydroxy3,6-dimethylbenzoate were active against *Candida albicans* (MIC:64 µg/mL) [65], while 2-ethylhexyl-4-methoxy orsellinate showed a notable effect against *Candida albicans* (MIC: 0.125 µg/mL) [65]. Among the lichens metabolites of (+) montagnetol homologs, (+) montagnetol homologs 3 exhibited excellent antimicrobial efficacy against *Pseudomonas aeruginosa* (MIC: 0.062 µg/mL), while (+) montagnetol homologs 6 significantly inhibited *Candida albicans* (MIC: 0.062 µg/mL); the C-2 and C-3 positional configurations of these compounds may be the key to the enhanced activity [66], which further highlighted the potential of lichens’ monocyclic derivatives in the antimicrobial field (Table 2).

#### 3.1.2. Depsides

Depsides, the core compounds of lichen acids, link multiple aromatic rings through ester bonds, which not only demonstrates chemical diversity but also showcases their outstanding antimicrobial activity [62]. Depside compounds from lichens, such as chloroatranorin [70], anziaic acid and its methylated derivatives [71], and barbatic acid and its derivatives [21] have been shown to have significant antimicrobial activity against *Staphylococcus aureus*, *Escherichia coli*, *Mycobacterium*, *Fusarium fujikuroi*, drug-resistant strains, and *Candida albicans*, among others, and have shown significant antibacterial activity. Among them, diffractaic acid (MIC: 16.3 µg/mL) showed higher antifungal activity than fluorocytosine (MIC: 90 µg/mL) and itraconazole (MIC: 27 µg/mL) [47]. Evernic acid showed significant antimicrobial activity, and its bacterial neuraminidase inhibitory activity was superior to quercetin [72], and divaricatic acid has stronger activity against *Staphylococcus epidermidis* and *Enterococcus faecium* (MIC: 16 µg/mL) than vancomycin (MIC: 25 µg/mL) [28]. Atranorin, derived from lichens such as *Cladonia foliacea* [30], *Usnea laevis* [23], *Menegazzia terebrata* [73], *Parmelia reticulate* [27], *Usnea rubrotincta*, and *Ramalina dumeticola* [53], showed significant activity against *Proteus vulgaris* (MIC: 5 µg/mL) and *Candida albicans*, comparable with erythromycin (MIC: 5.1 µg/mL) [74] and benomyl [21]. Perlatolic acid showed significant activity against methicillin-resistant *Staphylococcus aureus* (MIC: 32 µg/mL) and can act synergistically with gentamicin [75]. Lecanoric acid and olivetoric acid in this group demonstrates broad-spectrum antimicrobial activity [76]. Gyrophoric acid is particularly effective against *Bacillus subtilis* (MIC: 19 µg/mL) [62], and its unique structure with three monocyclic aromatic rings has been found in various lichens, including *Usnea muhlenbertus* [77], *Parmotrema tinctorum* [78], and *Acarospora fuscata* [21]. The activities of these compounds are closely related to their chemical structures, especially the compounds containing free phenolic groups, show strong inhibitory activities against Gram-negative bacteria [79], which is important in the search for effective anti-Gram-negative drugs (Table 3). Overall, depside compounds occupy a crucial position among the antimicrobially active compounds in lichens due to their unique chemical structural diversity and excellent biological activities.

#### 3.1.3. Depsidones

Depsidones, an important branch of *β*-type lichen phenolic compounds, exhibit remarkable antimicrobial properties [73]. In this category, psoromic acid was more effective (MIC: 3.2–4.1 μM) than isoniazid against *Mycobacterium tuberculosis* [82], while salazinic acid and lobaric acid also demonstrate significant inhibitory effects against drug-resistant *Mycobacterium tuberculosis* (MDR-R, MDR-40) (MIC: 50 μg/mL) [23]. In the field of oral health, variolaric acid, psoromic acid, hypoprotocetraric acid, and conhypoprotocetraric acid effectively inhibited the oral microorganisms *Streptococcus gordonii* and *Porphyromonas gingivalis*, with psoromic acid having the most significant activity, with MIC values of 11.72 μg/mL and 5.86 μg/mL, respectively [64]. For *Staphylococcus aureus*, lobaric acid (MIC: 8 μg/mL) showed strong activity [74], and protocetraric acid (MIC: 12.5 μg/mL) [83], psoromic acid (MIC: 31 μg/mL) [82] and himantormione A and B [69] were also active against this bacterium. Additionally, protocetraric acid demonstrate excellent antimicrobial activity against Gram-positive bacteria [84] and fungi of *Candida* (MIC: 3.9 μg/mL) [83]. Stictic acid [59] and norstictic acid [47] show excellent antimicrobial activity against *Francisella tularensis* and *Fusarium fujikuroi* among other microorganisms. Physodic acid [54], 3-hydroxyphysodic acid [59], and fumarprotocetraric acid possessed a wide range of microorganisms, with fumarprotocetraric acid showing outstanding activity against *Bacillus species*, *Listeria monocytogenes* (MIC: 4.6 μg/mL), and *Candida* fungi (MIC: 18.7 μg/mL) (Table 4) [30]. These findings indicate that depsidones show significant potential in both the antibacterial and antifungal fields, and their research and development are of notable scientific interest.

#### 3.1.4. Dibenzofuran Derivatives

Lichens produce unique dibenzofuran compounds through the polyketide pathway that are synthesized from phenolic units, forming aromatic or saturated derivatives rarely found in organisms outside of lichens [86]. Usnic acid, as a typical representative of this class of compounds, appears in various lichens in different enantiomeric forms, possibly as pure or mixed forms [87], exhibiting broad antimicrobial potential and effectively combating Gram-positive bacteria, such as *Mycobacterium abscessus* (MIC: 9.07/18.15 µg/mL) [88] and drug-resistant *Mycobacterium tuberculosis* (MIC: 12.5/25 µg/mL) [23] and many other microorganisms. It also showed significant inhibitory effects against *Staphylococcus aureus*, *Bacillus subtilis*, and *Clavibacter michiganensis* subsp. *Michiganensis* (MIC: 7.81 µg/mL) [53]. In the antifungal field, usnic acid showed potent activity against *Saprolegnia* (MIC: 2–8 µg/mL), especially against *Saprolegnia parasitica* [46], and inhibited *Candida albicans* (MIC: 0.25 µg/mL) and *Aspergillus fumigatus* (MIC: 0.125 µg/mL) [84]. Usnic acid derivatives, especially compounds containing cyclic sulfonamides, showed potent activity against *Mycobacterium tuberculosis* (MIC: 2.5–5.4 µM) [89]. The introduction of fluorine atom enhances its antimicrobial effect; e.g., 3-fluoro-5-trifluoromethylphenyl was effective against various pathogenic bacteria (MIC: 10 µM) [90]. The enantiomeric form also affects the antimicrobial activity of lichen compounds, as shown by the two enantiomers of usnic acid, (+)-usnic acid and (−)-usnic acid, which exhibit different antimicrobial actions; (+)-usnic acid was found to be effective against *Staphylococcus epidermidis* (MIC: 2.95 µg/mL) comparable to vancomycin (MIC: 3.12 µg/mL) [91], while (−)-usnic acid was more effective against *Staphylococcus aureus* (MIC: 2.4 µg/mL) [30]. In addition, other compounds in this group such as usenamine and its derivatives also exhibit broad antimicrobial effects. Usenamine E~H effectively inhibit *Candida albicans* (MIC: 64 µg/mL) [65], and usone fought against *Trichophyton rubrum*, a fungus causing skin infections (MIC: 41 µM) [92]. In terms of antibacterial activity, usnic acid is also effective in inhibiting *Escherichia coli* (MIC: 0.25 µg/mL) [38], *Mycobacterium tuberculosis* (MIC: 50 µg/mL) [23], *Klebsiella pneumoniae* (MIC: 0.0625 µg/mL) [84], and other pathogenic bacteria (Table 5). In conclusion, the dibenzofuran derivatives in lichens, with their broad antimicrobial properties, demonstrate substantial potential against Gram-positive bacteria, such as tuberculosis, *Staphylococcus aureus* and *Bacillus subtilis*.

#### 3.1.5. Other Phenol Derivatives

Phenolic acid derivatives of lichens, including xanthone and anthraquinone, exhibit promising antimicrobial activity. The primary structure of xanthone is 9H-xanthen-9-one with a dibenzo-γ-pirone scaffold [100], characterized by the internal cyclization of a single folded polyketone chain [101], and it is widely distributed in nature, with lichen-derived xanthones accounting for 79% of the total amount [102]. Lichen oxyxanthone chloride are of interest for their antibacterial and antifungal activities. It has been shown that the substitution of chlorine atom at the C-position significantly enhances its antimicrobial activity, e.g., 3-chloro-4,6-dimethoxy-1-methyl-9H-xanthen-9-one with a chlorine atom at the C-3 position showed antimicrobial activity against *Enterococcus faecalis* (IZ:10 mm) and *Staphylococcus aureus* (IZ:9.5 mm). Meanwhile, 2,7-Dichloro-3,4,6-trimethoxy-1-methyl-9H-xanthen-9-one with chlorine atoms at both C-2 and C-7 positions exhibited potent antifungal activity, especially against clinical dermatophytes, such as *Trichophyton rubrum*, *Microsporum canis*, and *Epidermophyton floccosum*, with MIC values ranging from 4 to 8 µg/mL and showed synergistic effects against *Trichophyton rubrum* in combination with fluconazole (FICI = 0.289) [102]. Anthraquinone derivative parietin exhibited antimicrobial activity against various bacterial strains, especially against *Staphylococcus aureus* and *Enterococcus faecalis* (MIC: 7.8–62.5 µg/mL), and also showed significant effects against *Rhizoctonia solani* (MIC: 31.3 µg/mL) [39]. Other compounds with antibacterial properties, such as lepraric acid, effectively combat oral pathogenic bacteria like *Porphyromonas gingivalis* and *Streptococcus gordonii* [64], and eumitrins F–H showed moderate inhibition against various microorganisms (MIC: 62.5 µg/mL) [103]. Hybocarpone exhibited notable antibacterial effect against *Staphylococcus aureus* and its methicillin-resistant *Staphylococcus aureus* strain (MIC: 4–8 µg/mL) (Table 6) [74]. Therefore, it is scientifically important to deeply explore the antimicrobial potential of other phenolic acids in lichens.

### 3.2. Higher Fatty Acids and Esters

Higher fatty acids and esters in lichens have also been confirmed to exhibit significant antimicrobial properties. For example, protolichesterinic acid from *Cetraria islandica* has a broad antibacterial and antifungal spectrum, such as methicillin-resistant *Staphylococcus aureus* (MIC: 64 µg/mL) [75] and *Pythium debaryanum* (ED_50_: 16.07 µg/mL), with activity against the latter exceeding that of hexaconazole (ED50: 25.92 µg/mL) [27]. Constipatic acid and 18r-ydroxy-dihydroalloprotolichesterinic acid from *Usnea* showed antifungal activity against *Candida albicans* (MIC: 64 µg/mL) [65]. The butyrolactone derivatives of lichesterinic acid, especially B-12, demonstrated excellent inhibitory effects against *Porphyromonas gingivalis* (MIC: 0.037 µg/mL) due to its COOH group and long carbon chain structure [104], showing promising application prospects in new drug discovery and oral care products and good prospects for application in new drug development and oral care products (Table 7).

### 3.3. Other Categories

Lichens, as pioneer organisms in nature, not only produce structurally distinctive compounds but also harbor numerous secondary metabolites with significant antimicrobial activities through the shikimic acid and mevalonic acid pathways, including triphenylquinone, picrotoxinin derivatives, and terpenoids, which provide important options for novel antimicrobial drug development [59]. In the area of antibacterial activity against Gram-positive bacteria, rhizocarpic acid (MIC: 32 µg/mL) [105] and caperatic acid (MIC: 10 µg/mL) [96] inhibited *Staphylococcus aureus*, while epiforellic acid showed inhibitory effects against methicillin-resistant *Staphylococcus aureus* (MIC: 32 µg/mL) [75]. Vulpinic acid, derived from *Letharia vulpina* [106], inhibited not only methicillin-resistant *Staphylococcus aureus* and oral pathogenic *Streptococcus gordonii* (MIC: 187.5 µg/mL) and *Porphyromonas gingivalis* (MIC: 375 µg/mL) [64] but also strongly inhibited the phytopathogenic fungus *Sclerotinia sclerotiorum* (EC_50_: 2.8 µg/mL), revealing its potential application in plant disease management [76]. Moreover, stereocalpin A (IC_50_: 28 µg/mL), stereocalpin B (IC_50_: 30 µg/mL) [99] and uridine (IZ:6.3 mm) [65] exhibited antimicrobial activity against *Escherichia coli*, while 4-(acylamino) butyramides and (+)-roccellic acid showed activity against pathogenic *Candida albicans* (MIC: 64 µg/mL) [65] and the oral-associated bacteria *Streptococcus gordonii* and *Porphyromonas gingivalis* (MIC: 46.9 µg/mL) [64], thus offering rich natural resources for antimicrobial drug development (Table 8).

In the field of antiviral research, in addition to indicators such as IC_50_, ED_50_, and IR, the selectivity index (SI) is also an important evaluation criterion. A higher SI value indicates that the drug has lower toxicity to host cells while inhibiting the virus [109,110]. As illustrated in Table 9, several monocyclic derivatives, including methyl-β-orcinol carboxylate, atranol, and methyl haematommate, have been shown to exert inhibitory effects on the *hepatitis C virus*, with IC_50_ values ranging from 40.3 to 55.5 μM [111]. In addition, depsidic compounds from lichens have also demonstrated excellent antiviral activity. For example, evernic acid exhibited a suppression rate of 64.6% against the *Epstein–Barr virus* at a concentration of 50 µM, with no mutagenicity or tumorigenicity [62]. Atranorin showed promising inhibitory effects against the *hepatitis C virus* (IC_50_: 22.3 μM, SI > 4.5) [111]. Sekikaic acid exhibited significant inhibitory effects and selectivity against respiratory syncytial virus (IC_50_: 5.69 μg/mL, SI: 5.46) [62,112]. Depsidic compounds isolated from *Usnea longissima*, particularly barbatic acid, were proven to inhibit the neuraminidase of the influenza virus (IC_50_: 8.44 μM) [72]. Depsides, such as lobaric acid, demonstrated antiviral activity against chikungunya virus and the novel *Severe acute respiratory syndrome-related coronavirus* 2 (coronavirus SARS-CoV-2) [59]. Additionally, psoromic acid effectively inhibited the replication of *herpes simplex virus* (HSV) type 1 (IC_50_: 1.9 μM, SI: 163.2) and type 2 (IC_50_: 2.7 μM, SI: 114.8), with efficacy surpassing the antiviral drug acyclovir (IC_50_: 2.6 and 2.8 μM, SI: 119.2 and 110.7), indicating higher selectivity in inhibiting HSV [113]. Dibenzofuran derivatives, including usnic acid and its derivatives, also exhibited remarkable antiviral activity, effectively inhibiting the proliferation of mouse polyomavirus and showing antiviral activity against human papillomavirus and arenaviruses [114]. Among the isomers, for SARS-CoV-2, (+)-usnic acid (IC_50_: 7.99 µM, SI: 6.26) showed higher selectivity than chloroquine (IC_50_: 6.16 µM, SI: 13.07) and remdesivir (IC_50_: 7.42 µM, SI: 4.24) but lower selectivity than lopinavir (IC_50_: 10.8 µM, SI: 6.74) [114,115], demonstrating some selective advantage, but with room for improvement. In terms of inhibiting viral activity, (+)-usnic acid exhibited greater inhibitory effects against SARS-CoV-2 than remdesivir [114]. Furthermore, (+)-usnic acid achieved a selectivity index of 11.1 against the Beta variant (B.1.351) of SARS-CoV-2, which is higher than that of the Alpha variant (B.1.1.7, SI: 5.8), indicating lower toxicity to host cells when inhibiting the Beta variant [115]. Regarding the inhibition of the A (H1N1) pdm09 *influenza virus*, (−)-usnic acid had a selectivity index of 14.4, higher than that of (+)-usnic acid (SI: 5.9), showing greater selectivity [87,116]. Additionally, usnic acid derivatives inhibited the growth of several influenza viruses, such as H1N1pdm, H3N2, A/Vladivostok/2/09 (H1N1), and *influenza A virus* (Puerto Rico/8/1934, H1N1), with IC_50_ values ranging from 3 to 43 µg/mL [114]. In conclusion, psoromic acid, (+)-usnic acid, and (−)-usnic acid all exhibit good antiviral activity and selectivity against HSV, SARS-CoV-2 and its variants, as well as the A (H1N1) pdm09 *influenza virus*, indicating potential for further research and development.

## 4. Potential Applications and Challenges of Lichen Antimicrobial Activity

### 4.1. Application Value of Lichen Antimicrobial Activity

Lichens, as a unique biological resource, demonstrate immense potential in various fields such as the medicine, agriculture, and food industries due to their rich chemical composition and diverse biological activities. In the field of pharmaceuticals, lichen-derived compounds, especially usnic acid and its derivatives, excel in the antimicrobial field, with remarkable efficacy against various skin infections and skin diseases [95], and have been widely used in facial infections, ulcers, burns, and scars [32,117]. Lichens such as *Lobaria pulmonaria*, *Cetraria islandica*, and *Cladonia* species are used for the treatment of tuberculosis, and *Cetraria islandica* in particular is famous in Turkey for its therapeutic effects on hemorrhoids, pneumonia, and dysentery [118]. Additionally, *Xanthoria parietina*, *Letharia vulpine*, and *Parmelia sulcata* are used for the treatment of jaundice, digestive system disorders, and respiratory disorders, respectively [118]. These lichens occupy a significant place in traditional medicine due to their unique medicinal value. In modern medicine, the application of usnic acid has expanded to antimicrobial coatings for medical devices [95] and polymeric materials [87], such as usnic acid polyaniline matrix dressings Fe_3_O_4_@AU [119,120], polymethylmethacrylate (PMMA) bone cements [119], titanium implants, and polymeric implants for tympanic membranes [121], which can effectively reduce bacterial biofilm formation and enhance the antimicrobial properties of medical devices. Usnic acid is also used in personal care and hygiene products such as dandruff and itching shampoo, medical mouthwashes, medical gloves, and disinfectants due to its remarkable antimicrobial activity [95]. Among these, usnic acid preparations, such as Sodium usnate and Copper (II) usnate [95], are widely used internationally and have demonstrated favorable clinical effects. Through chemical structural modifications, usnic acid derivatives possess multifunctional properties, such as antimicrobial and antiviral activities. For example, modifying its C-2 group to enamine can synthesize 1, 2, 3-triazole antimicrobial and antituberculosis agents [89]. The zinc salt of usnic acid has shown pharmacological effects in the treatment of various viral infections, such as *Human Papillomavirus* and *Influenza virus* [50]. In summary, usnic acid and its derivatives in lichens not only hold a significant place in traditional medicines but also play a key role in the antimicrobial treatment of medical devices and the development of wound dressings, which promotes the discovery of new medicines and the innovation of medical devices.

Meanwhile, lichens are also emerging in the field of agriculture; lichen extracts can inhibit the growth of plant pathogenic microorganisms and serve as natural plant protection agents for the prevention and control of crop diseases. Usnic acid, a representative compound of lichens extracts, can efficiently suppress pathogenic *Oomycetes*, aiding in the control of saprolegniasis in aquaculture [46]. Beard lichen extract has therapeutic effects on rainbow trout infected with *Lactococcus garvieae* [122], which contributes to microbial pollution control in aquatic ecosystems [44]. Additionally, the antifungal activity of usnic acid and vulpinic acid provides significant control of bacterial canker of tomato [123]. *Trichoderma asperellum* has been shown to be effective in the control of ryegrass brown patch caused by *Rhizoctonia solani* on golf courses, making it a promising candidate for new biopesticides [76].

In the food industry, the application potential of lichen extracts is equally notable. *Oakmoss* lichen is used to make jelly, and *Cladonia rangiferina* is used in brandy production, enriching the flavor of foods while offering potential health benefits [118]. Usnic acid is used as a nutraceutical ingredient in some countries to induce weight loss [115], though excessive intake may lead to hepatotoxicity and acute failure [124]. Usnic acid is also an efficient cream preservative with strong inhibitory effects on a wide range of microorganisms in thin cream [20]. Moreover, extracts of *Usnea barbata* [50], *Parmelia saxatilis* [27], and their zinc salts, highly sensitive to *Enterococcus*, became natural feed additives for poultry. In the field of food packaging materials, thin coatings of lichens based on ZnO@C18-usnic acid nanoparticles were prepared by MAPLE technology, which effectively inhibited the adhesion and biofilm formation of *Salmonella*, offering an innovative choice for new food packaging materials [125]. It is also noteworthy that lichens compounds have industrial potential for the preparation of PH indicators [27], dyes [27], daily products such as toothpaste and mouthwash [115], UV protectants, or sunscreens [27]. For example, the thallus of *Evernia prunastri* and *Usnea* is used in perfume production, while Wolf lichen is a widely used purple dye used by North American indigenous people [118].

### 4.2. Challenges in the Application of Lichen Antimicrobial Activity

Despite the promising applications of lichens as potential antimicrobial agents, several challenges hinder their practical application in the medical field and in functional foods. The primary issue is the limited availability of raw materials for research and application due to constraints in algal physiology and CO_2_ diffusion [126], which result in slow natural growth, low biomass, and restricted access to lichen resources [16]. Moreover, the yield of active ingredients from lichens is highly dependent on environmental conditions [13,127]. For example, the production of secondary metabolites from lichens is unstable, influenced by various factors such as light, temperature, humidity, and altitude. Although artificially simulating the growth environment of lichens and optimizing controlled laboratory conditions could be a strategy to obtain sufficient amounts of active lichen feedstock, achieving this in a short timeframe may prove challenging [127]. To address this issue, researchers might explore expanding fermentation technologies for lichen endophytes, investigating active products derived from the fermentation liquid of these microorganisms to tackle the slow growth and scarcity of wild resources. In conjunction with the OSMAC strategy, new culture media and nutrient regulation techniques can be developed to activate silent gene clusters by modulating nutritional or environmental factors during fermentation, thereby increasing the yield of secondary metabolites or acquiring similar efficient compounds [16,128]. Additionally, multi-omics technologies, such as genomics, transcriptomics, proteomics, and metabolomics, can be employed to analyze the metabolic pathways of lichen endophytes [129]. Coupled with gene scanning and gene editing technologies like CRISPR/Cas9 [130], this approach facilitates precise modifications of key metabolic pathways, potentially increasing both the yield and stability of secondary metabolites [11]. This process includes activating and regulating the expression of various functional genes, optimizing key enzymes in metabolic pathways, and ensuring a stable supply of raw materials while also potentially leading to the discovery of new compounds with novel structures and broad biological activities [131]. Furthermore, biosynthetic methods such as microbial metabolic engineering and plant transformation can be utilized to enhance the catalytic efficiency of key enzymes through enzyme engineering [132], optimizing the “cell factory” and fermentation conditions (including enzyme catalytic efficiency and substrate supply), thereby increasing the yield and conversion rate of target compounds and promoting the sustainable use of lichen resources [133]. Through these technologies, researchers can identify and optimize key enzymes and regulatory factors via metabolic engineering, ultimately enhancing yields of secondary metabolites [134]. This strategy effectively addresses the challenges associated with the limited availability of lichen resources and the instability of secondary metabolite yields while also promoting the sustainable utilization of lichen resources and providing a vital research foundation for new drug development.

Secondly, the complexity of lichen taxonomy poses challenges for the identification of lichen species and their products. To address this issue, it is recommended to adopt an integrated multidisciplinary approach to identification, including molecular biology techniques, chemical analysis methods, and morphological observations, to ensure an accurate classification of lichens and provide a solid foundation for research and application [135,136]. Additionally, utilizing advanced microscopic imaging technologies to observe the morphological and structural characteristics of lichens, combined with chemical analysis results, can further improve the accuracy of species identification [137]. Moreover, constructing molecular networks of lichen metabolites to analyze the interrelationships among metabolites may help uncover new metabolic pathways and potential bioactive compounds [16]. In this process, establishing a simple yet scientific identification system is crucial for researchers, as it will contribute to the standardization and efficiency of lichen research.

Thirdly, the potential toxicity of lichen metabolites and photosensitization have limited their applications in the pharmaceutical field [21,80]. To address this issue, a variety of innovative strategies can be employed: Computer-aided drug design (CADD) can be utilized to optimize the structures of lichen metabolites, screening for derivatives with higher selectivity and lower toxicity, thereby predicting activity and toxicity at the molecular level and guiding the synthesis of safer compounds [138]. Additionally, microbial transformation techniques can harness the metabolic capabilities of microorganisms to convert toxic compounds into low-toxicity or non-toxic derivatives while preserving their biological activity [139]. Furthermore, a combination therapy strategy is also an effective approach; co-administration with other drugs can alleviate allergic reactions caused by lichen metabolites like usnic acid [126]. Moreover, developing smart drug delivery systems, such as nanocarriers and targeted drug delivery technologies, can effectively reduce the adverse effects of drugs and improve treatment safety and efficacy [20,126]. Examples include nanogels [140], peptoids [141], liposomes [142], and CBD-loaded PEG-b-PCL nanoparticles, the latter of which have been used in drug nanocarriers due to their excellent biocompatibility and have been approved by the FDA [143]. Polymer carriers such as Risperdal Consta^®^, Trelstar^®^, Sandostatin LAR^®^, and Somatuline Autogel^®^ have the ability to precisely deliver drugs to specific cells or organelles, enhancing drug efficacy and reducing side effects [142]. Additionally, pro-drug strategies can chemically modify active drugs into inactive or low-activity forms that release active compounds under specific conditions in the body, thereby reducing direct toxicity while increasing targeting and bioavailability [144]. Finally, gene editing technologies can be used to optimize the metabolic pathways of lichen endophytes or symbiotic fungi, thereby reducing the generation of toxic metabolites at the source [130]. The comprehensive application of these strategies not only effectively reduces the potential toxicity of lichen metabolites but also enhances their value in the pharmaceutical field, providing broader prospects for the development of safe and effective lichen-derived drugs.

Fourthly, the structural complexity of lichen secondary metabolites increases the difficulty of extraction, purification, and identification, leading to high costs. To address these challenges, a variety of innovative strategies can be employed. First, it is recommended to use techniques such as thin-layer chromatography [145], capillary gas chromatography [146], silica gel column chromatography, medium-pressure liquid chromatography [147], supercritical fluid extraction, and high-performance liquid chromatography [146] to improve efficiency and purity. In addition, the use of computational chemistry and chemical biology to simulate and predict the chemical properties of these molecules can help accelerate their identification and functional research [148]. Furthermore, utilizing gene-editing techniques to precisely modify lichen endophytes or symbiotic fungi can optimize metabolic pathways, reduce the production of complex metabolites, and enhance the yield of target compounds [130]. Implementing automation equipment for rapid sample processing and large-scale screening not only reduces human error but also lowers research costs [149]. Finally, optimizing the solvent system (for example, developing more efficient solvent combinations) can further improve the resolution of thin-layer chromatography and other separation techniques [144]. The comprehensive application of these strategies will significantly enhance the extraction efficiency and purity of lichen secondary metabolites, reduce research costs, and provide strong support for the sustainable utilization of lichen resources and the development of new drugs.

Fifthly, the commercialization and practical translation of lichen active compounds may face market and regulatory barriers. Like other novel antibiotics, the development of new lichen drugs encounters challenges in market access and regulatory approval, which may delay their actual application [21]. To overcome this challenge, researchers should strictly adhere to regulatory requirements for drug development and conduct comprehensive safety and efficacy assessments from the early research stages. Meanwhile, they should engage in active communication with government and regulatory agencies to seek policy support and fast-track approval processes to shorten the time to market [21,150]. Additionally, through international collaboration and regulatory coordination, introducing advanced international research and development experiences and optimizing the import approval process can facilitate the international development of lichen drugs [21,150]. Utilizing advanced clinical trial designs and data analysis methods can improve the efficiency and success rate of clinical trials [21,150] while also promoting the informatization of production and inspection processes in pharmaceutical companies, enhancing the transparency and controllability of drug production. These measures will help accelerate the commercialization process of lichen active compounds and facilitate their transition from the laboratory to the market.

Sixthly, the economic cost of lichens and their metabolic product production and development is a significant challenge. Based on the aforementioned difficulties, the production and development of lichens and their metabolites are costly, which to some extent affects their commercialization prospects [16]. It is suggested that lichen researchers should seek diversified financial support, such as governmental research funds, corporate investment and international cooperation, etc., so that the target compounds can be synthesized on a large scale by means of synthetic biology after obtaining highly promising antimicrobial active substances [151].

Seventhly, lichen extracts have demonstrated significant antibacterial activity in in vitro experiments. However, most current research remains in the preclinical stage, primarily focusing on in vitro studies and animal models, lacking supportive data from large-scale clinical trials [21]. Additionally, while compounds like usnic acid have found some applications in everyday products [95] such as health supplements, cosmetics, toothbrushes, antimicrobial coatings [95], and food packaging [125], issues related to their toxicity and bioavailability have not yet been fully resolved, and they are not currently used as standalone drugs for clinical treatment. Furthermore, there are currently no clinical data indicating that patients can directly use lichens or their compounds to treat infectious diseases, suggesting that the application of lichens and their compounds is still in the experimental exploration stage and has not yet transitioned into clinical practice [21]. Future research should focus on clinical trials of lichen compounds to determine their potential application value in treating infectious diseases [29]. At the same time, the research on their mechanisms of action is still at an early stage, with unclear molecular targets and pathways [152]. Limited experimental data and insufficient clinical validation also restrict their promotion in practical applications [21]. Moving forward, it is important for researchers in this field to utilize modern technological methods to explore their mechanisms of action [16], improve experimental data, and conduct clinical validations to overcome regulatory hurdles and advance their application in both medical and industrial fields.

Finally, the issue of public acceptance cannot be ignored. On the one hand, the public has limited understanding of lichens, and on the other hand, cases of adverse reactions or even deaths did occur during the transformation process of existing lichen active substance applications, causing public doubts about the safety of lichen-derived drugs [20]. For this reason, more scientists are needed to join lichen research teams and carry out extensive widespread public education and outreach to improve public awareness of lichens and their application potential. At the same time, their safety and effectiveness can be demonstrated through clinical trials and practical application data to enhance public trust [20].

Overall, lichens—these “antimicrobial warriors” hidden deep in nature—still face numerous global challenges on the road to widespread application in the pharmaceutical field, particularly during the critical phase of transitioning from the laboratory to clinical application. This process is complex and arduous, requiring not only rigorous clinical trials and practical application data to thoroughly demonstrate their safety and efficacy but also the ability to meet stringent market access and regulatory approval requirements. These challenges will undoubtedly delay the development process of lichen-based pharmaceuticals. However, even in the face of these obstacles, lichens and their unique secondary metabolites remain an important resource in the field of new drug development due to their exceptional antibacterial potential. We fervently call upon more scholars to engage in lichen research, delve deeply into the scientific issues mentioned above, and uncover the mysteries of this ancient organism.

## Figures and Tables

**Table 2:** Antimicrobial activity of monocyclic derivatives.

Compounds	Structures	Object Strains	Samples	Positive Control	References
			MIC (µg/mL)/IZ (mm)		
4-Chlororcinol	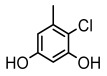	Methicillin-resistant *Staphylococcus aureus*	MIC: 1–17	Teicoplanin (MIC: 1)	[35]
		*Enterococcus faecalis*	MIC: 75	Teicoplanin (MIC ≤ 0.5)	
		*Acinetobacter baumannii*	MIC: 300	Colistin (MIC: 0.78)	
		*Klebsiella pneumoniae*		Colistin (MIC < 2)	
Orcinol	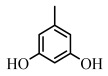	*Enterococcus faecium*	MIC: 9.37	Teicoplanin (MIC ≤ 0.5)	[35]
		Methicillin-resistant *Staphylococcus aureus*	MIC: 18.75	Colistin (MIC: 1)	
		*Escherichia coli*	MIC: 9.37	Teicoplanin (MIC: 1)	
		*Pseudomonas aeruginosa*	MIC: 300	Colistin (MIC: 4)	
Methyl β-orcinol-carboxylate	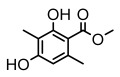	*Streptococcus gordonii*	MIC: 375	Doxycycline (MIC: 0.13)	[64]
		*Porphyromonas gingivalis*	MIC: 93.75	Doxycycline (MIC: 0.51)	
Orsellinic acid	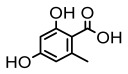	*Fusarium fujikuroi*	MIC: 15.1	Amphotericin B (MIC: 3) Isavuconazole (MIC: 5) Natamycin (MIC: 4) Posaconazole (MIC: 0.65) Voriconazole (MIC: 3.7) Fluconazole (MIC: 90) Itraconazole (MIC: 27)	[47]
Methyl β-orsellinate	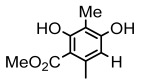	*Enterococcus faecium*	IZ: 13.0	Apramycin (IZ: 21.0)	[63]
		*Staphylococcus aureus*	IZ: 18.0	Apramycin (IZ: 21.0)	[67]
		*Staphylococcus aureus*	MIC: 62.5	Streptomycin (MIC: 2)	[68]
		*Bacillus subtilis* *Bacillus cereus*	MIC: 125	Streptomycin (MIC: 4)	
		*Staphylococcus epidermidis*		Streptomycin (MIC: 2)	
		*Acinetobacter baumannii*	IZ: 16.0	Apramycin (IZ: 20.0)	[63]
		*Helicobacter pylori*	IZ: 27.0		[44]
		*Escherichia coli*	MIC: 62.5	Streptomycin (MIC: 4)	[68]
		*Shigella sonnei*	MIC: 125		
Methyl 5-bromo-β-orsellinate	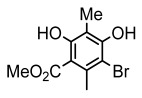	*Staphylococcus aureus*	IZ: 12.0		[67]
Methyl 3,5-dibromo-orsellinate	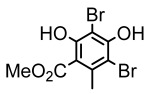	*Staphylococcus aureus*	IZ: 29.0 MIC: 4		[67]
Ethyl everninate	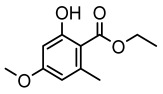	*Candida albicans*	MIC: 64		[65]
Atranol	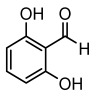	*Staphylococcus aureus*	IC_50_ ≥ 200,000	Kanamycin (IC_50_: 42)	[69]
		*Escherichia coli*		Kanamycin (IC_50_: 9)	
Dibutyl phthalate	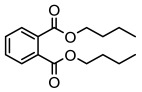	*Candida albicans*	MIC: 64		[65]
Methyl orsellinate	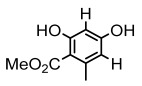	*Staphylococcus aureus*	IZ: 13.0		[67]
		*Helicobacter pylori*	IZ: 22.0		[44]
Orsellinaldehyde	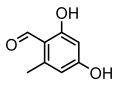	*Staphylococcus aureus*	IZ: 6.6	Gentamicin (IZ: 12.3)	[65]
Methyl-2,4-dihydroxy3,6-dimethylbenzoate	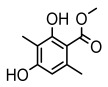	*Candida albicans*	MIC: 64		[65]
2-Ethylhexyl-4-methoxy orsellinate	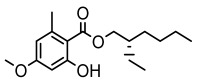	*Staphylococcus aureus*	IZ: 6.2	Gentamicin (IZ: 12.3)	[65]
		*Escherichia coli*	IZ: 6.3	Gentamicin (IZ: 12.4)	
(+) Montagnetol	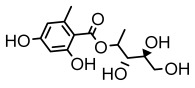	*Staphylococcus aureus*	MIC: 0.5	Streptomycin (MIC: 0.007)	[66]
		*Salmonella typhi Pseudomonas aeruginosa*	MIC: 0.25	Streptomycin (MIC: 0.015)	
		*Escherichia coli*	MIC: 0.5	Streptomycin (MIC: 0.125)	
		*Candida albicans*	MIC: 0.125	Streptomycin (MIC: 0.031)	[66]
(−) Montagnetol	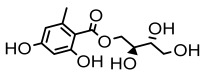	*Staphylococcus aureus*	MIC: 0.5	Streptomycin (MIC: 0.007)	[66]
		*Salmonella typhi*	MIC: 0.25	Streptomycin (MIC: 0.015)	
		*Escherichia coli*		Streptomycin (MIC: 0.125)	
		*Pseudomonas aeruginosa*	MIC: 0.5	Streptomycin (MIC: 0.015)	
		*Candida albicans*	MIC: 0.125	Streptomycin (MIC: 0.031)	
		*Staphylococcus aureus*	MIC: 0.125	Streptomycin (MIC: 0.007)	[66]
(+) Montagnetol homologs 3	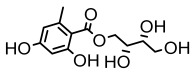	*Salmonella typhi*	MIC: 0.125	Streptomycin (MIC: 0.015)	
		*Pseudomonas aeruginosa*	MIC: 0.062	Streptomycin (MIC: 0.015)	
		*Escherichia coli*	MIC: 0.5	Streptomycin (MIC: 0.125)	
		*Staphylococcus aureus*	MIC: 0.5	Streptomycin (MIC: 0.007)	[66]
(+) Montagnetol homologs 6	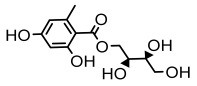	*Salmonella typhi Pseudomonas aeruginosa*	MIC: 0.25	Streptomycin (MIC: 0.015)	
		*Escherichia coli*		Streptomycin (MIC: 0.125)	
		*Candida albicans*	MIC: 0.062	Streptomycin (MIC: 0.031)	

MIC: minimum inhibitory concentration; IZ: inhibition zone diameter.

**Table 3:** Antimicrobial activity of depsides.

Compounds	Structures	Object Strains	Samples	Positive Control	References
			MIC/EC_50_/ED_50_ (µg/mL)/IZ (mm)/IR (%)		
Chloroatranorin	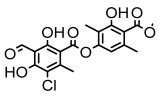	*Staphylococcus aureus*	MIC: 6240		[70]
		*Bacillus cereus*			
		*Bacillus subtilis*			
		*Listeria monocytogenes*	MIC: 3120		[70]
		*Proteus vulgaris*	MIC: 6240		
		*Aeromonas hydrophila*	MIC: 3120		
		*Yersinia enterocolitica*	MIC: 6240		
		*Candida albicans*	MIC: 12,520		[70]
		*Candida glabrata*			
Anziaic acid	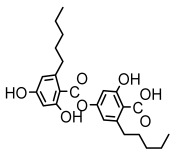	*Bacillus cereus*	MIC: 500	Streptomycin (MIC: 16)	[71]
2′-O-Methyl anziaic acid	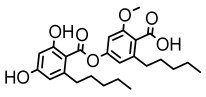	*Bacillus cereus*	MIC: 62.5	Streptomycin (MIC: 16)	[71]
		*Staphylococcus aureus*	MIC: 250	Streptomycin (MIC: 31)	
		*Escherichia coli*	MIC: 1000	Streptomycin (MIC: 62)	[71]
		*Proteus mirabilis*	MIC: 500	Streptomycin (MIC: 62)	
		*Cladosporium cladosporioides*	MIC: 250	Ketoconazole (MIC: 39)	[71]
		*Candida albicans*			
		*Trichoderma viride*	MIC: 250	Ketoconazole (MIC: 78)	
		*Fusarium oxysporum*			
		*Alternaria alternate*			
		*Mucor mucedo*	MIC: 500	Ketoconazole (MIC: 156)	
		*Penicillium expansum*			
		*Aspergillus niger*	MIC: 500	Ketoconazole (MIC: 78)	
		*Penicillium chrysogenum*			
		*Aspergillus flavus*	MIC: 1000	Ketoconazole (MIC: 312)	
Barbatic acid	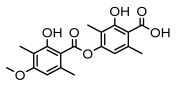	*Bacillus subtilis*	MIC: 31.25	Chloramphenicol (MIC: 7.81) Vancomycin (MIC: 7.81)	[53]
		*Staphylococcus aureus*	MIC: 62.5	Chloramphenicol (MIC: 31.25) Vancomycin (MIC: 15.63)	
4′-O-Demethylbarbatic acid	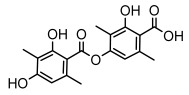	*Streptococcus gordonii*	MIC: 218	Doxycycline (MIC: 0.51)	[64]
		*Porphyromonas gingivalis*	MIC: 10.94	Doxycycline (MIC: 0.13)	[38]
3-Hydroxy-5methylphenyl-2-hydroxy-4-methoxy-6-methylbenzoate	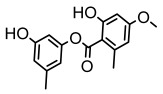	*Staphylococcus aureus*	IZ: 6.6	Gentamicin (IZ: 12.3)	[65]
		*Candida albicans*	MIC: 32		[65]
Diffractaic acid	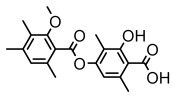	*Staphylococcus aureus*	IZ: 17.3		[62]
		*Mycobacteria*	MIC: 15.6		[47]
		*Escherichia coli*	IZ: 12.8		[62]
		*Fusarium fujikuroi*	MIC: 16.3	Amphotericin B (MIC: 3) Isavuconazole (MIC: 5) Natamycin (MIC: 4) Posaconazole (MIC: 0.65) Voriconazole (MIC: 3.7) Fluconazole (MIC: 90) Itraconazole (MIC: 27)	[47]
Diffractic acid	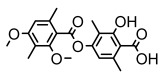	*Staphylococcus aureus*	IZ: 17.3	Amoxicillin (IZ: 22.0) Chloramphenicol (IZ: 30.8)	[48]
		*Escherichia coli*	IZ: 12.8	Amoxicillin (IZ: 15.8) Chloramphenicol (IZ: 31.2)	
Evernic acid	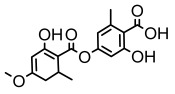	*Staphylococcus aureus*	MIC: 0.98		[62]
		*Staphylococcus aureus-1199B*	MIC: 128	Norfloxacin (MIC: 32)	[74]
		*Escherichia coli*	MIC: 31.25		[62]
		*Pseudomonas aeruginosa*	MIC: 125		
		*Candida albicans*	MIC: 62.5		[62]
8-Hydroxybarbatic acid	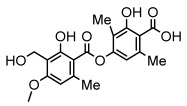	*Bacillus subtilis*	MIC: 125	Chloramphenicol (MIC: 7.81) Vancomycin (MIC: 7.81)	[53]
Methyl evernate	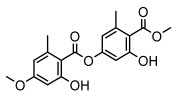	*Bacillus cereus*	MIC: 125	Streptomycin (MIC: 16)	[43]
		*Bacillus subtilis*	MIC: 250	Streptomycin (MIC: 16)	
		*Staphylococcus aureus*	MIC: 500	Streptomycin (MIC: 31)	
		*Escherichia coli*	MIC: 1000	Streptomycin (MIC: 62)	[43]
		*Proteus mirabilis*			
		*Candida albicans*	MIC: 250	Ketoconazole (MIC: 39)	[43]
		*Cladosporium cladosporioides*			
		*Alternaria alternate*		Ketoconazole (MIC: 78)	
		*Penicillium expansum Mucor mucedo*	MIC: 500	Ketoconazole (MIC: 156)	
		*Fusarium oxysporum Trichoderma viride*		Ketoconazole (MIC: 78)	
		*Penicillium chrysogenum Aspergillus niger*	MIC: 1000	Ketoconazole (MIC: 78)	
		*Aspergillus flavus*		Ketoconazole (MIC: 312)	
2′-Omethylevernol	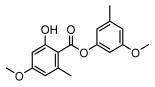	*Staphylococcus aureus*	IZ: 6.4	Gentamicin (IZ: 12.3)	[65]
		*Candida albicans*	MIC: 64		
Atranorin	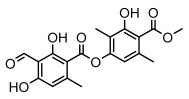	*Staphylococcus aureus-1199B*	MIC: 128	Norfloxacin (MIC: 32)	[74]
		*Staphylococcus aureus*	MIC: 31	Streptomycin (MIC: 31.25)	[30,53,80]
		Methicillin-susceptible *Staphylococcus aureus* (Sa1,Sa10,Sa13)	MIC: 128		[81]
		Methicillin-resistant *Staphylococcus aureus* (Sa3,Sa14)	MIC: 128		
		Methicillin-resistant *Staphylococcus aureus* (Sa15)	MIC: 64		
		*Bacillus mycoides*	MIC: 15~31	Streptomycin (MIC: 7.81)	[80]
		*Bacillus subtilis*	MIC: 15.63~70.7	Streptomycin (MIC: 7.81) Erythromycin (MIC: 4.2) Gentamycin (MIC: 5)	
		*Bacillus subtilis*	MIC: 15.6/15.63	Chloramphenicol (MIC: 7.81) Vancomycin (MIC: 7.81)	[30,53]
		*Sarcina lutea*	MIC: 21.5	Erythromycin (MIC: 4.6) Gentamycin (MIC: 4.5)	
		*Listeria monocytogenes*	MIC: 15.6		[30]
		*Streptococcus faecalis*	MIC: 250 IZ: 17.8~33.0	Erythromycin (MIC: 4) Gentamycin (MIC: 5)	[30,80]
		*Bacillus cereus*	MIC: 1.2		[30]
		*Mycobacterium tuberculosisuberculosis*	MIC: 250	Levofloxacin (MIC: 0.015)	[80]
		*Mycolicibacterium aurum*			
		*Mycobacterium tuberculosis* (MDR-A8)	MIC > 200	Rifampicin (MIC: 100)	[23]
		*Mycobacterium smegmatis* (MDR-40)			
		*Mycobacterium tuberculosis* (MDR-V791)	MIC > 200	Rifampicin (MIC > 200)	
		*Mycobacterium smegmatis* (MDR-R)			
		*Proteus vulgaris*	MIC: 5/62.5	Erythromycin (MIC: 5.1) Gentamycin (MIC: 4.6)	[30,80]
		*Aeromonas hydrophila*	MIC: 31.2		[30]
		*Escherichia coli*	MIC: 8.3~31	Streptomycin (MIC: 31.25) Erythromycin (MIC: 4.7) Gentamycin (MIC: 5.1)	[80]
		*Enterobacter cloacae*	MIC: 31/1000		
		*Klebsiella pneumoniae*	MIC: 8.3~31/500		
		*Candida glabrata*	MIC: 500		[30]
		*Candida albicans*	MIC: 17/250~500	Erythromycin (MIC: 5) Gentamycin (MIC: 4.9) Ketoconazole (MIC: 1.95)	[30,80]
		*Sclerotium rolfsii Sacc*	ED_50_: 39.70		[27]
		*Aspergillus fumigatus*	MIC: 250/500	Ketoconazole (MIC: 3.9)	[80]
		*Cryptococcus (Naganishia) diffluens*	MIC: 15.7	Erythromycin (MIC: 5.8) Gentamycin (MIC: 5.5)	[80]
		*Cryptococcus neoformans*	MIC > 250	Ketoconazole (MIC: 25)	[80]
		*Epidermophyton floccosum*			
		*Paecilomyces variotii*	MIC: 250	Ketoconazole (MIC: 1.95)	[80]
		*Trichoderma harzianum*		Ketoconazole (MIC: 7.81)	
		*Botrytis cinerea*		Ketoconazole (MIC: 1.95)	
		*Fusarium oxysporum*	MIC: 500	Ketoconazole (MIC: 3.9)	[80]
		*Mucor mucedo*		Ketoconazole (MIC: 31.25)	
		*Penicillium purpurescens*	MIC: 500~1000	Ketoconazole (MIC: 3.9)	[80]
		*Penicillium verrucosum*			
		*Aspergillus flavus*			
Divaricatic acid	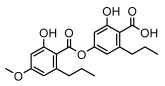	*Bacillus subtilis*	MIC: 7	Vancomycin (MIC: 0.78) Cefotaxime (MIC: 0.5)	[28]
		*Staphylococcus aureus 0027*	MIC: 64	Vancomycin (MIC: 25) Cefotaxime (MIC: 64)	
		*Staphylococcus epidermidis*	MIC: 16	Vancomycin (MIC: 25) Cefotaxime (MIC: 0.5)	
		*Enterococcus faecium*	MIC: 16	Vancomycin (MIC: 25) Cefotaxime (MIC > 256)	
		Methicillin-resistant *Staphylococcus aureus*	MIC: 30	Vancomycin (MIC: 25) Cefotaxime (MIC > 256)	
		*Streptococcus mutans*	MIC: 32	Vancomycin (MIC: 12.5) Cefotaxime (MIC: 0.5)	
		*Micrococcus luteus*	MIC: 40	Vancomycin (MIC: 25) Cefotaxime (MIC: 1)	
		*Pseudomonas aeruginosa*	MIC: 128	Vancomycin (MIC: 31.25) Cefotaxime (MIC: 32)	[28]
		*Candida albicans*	MIC: 20	Vancomycin (MIC > 100) Cefotaxime (MIC > 256)	[28]
Perlatolic acid	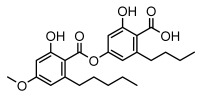	Methicillin-resistant *Staphylococcus aureus*	MIC: 32	Clindamycin (MIC: 8192) Erythromycin (MIC: 1024) Gentamicin (MIC: 256) Levofloxacin (MIC ≤ 0.5) Oxacillin (MIC: 8)	[75]
Thamnolic acid	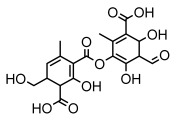	*Bacillus cereus*	MIC: 400		[62]
		*Bacillus subtilis*			
		*Listeria monocytogenes*	MIC: 200		
		*Micrococcus luteus*			
		*Proteus vulgaris*	MIC: 400		[62]
		*Sclerotium rolfsii Sacc*	MIC: 200		[62]
		*Candida krusei*	MIC: 400		
		*Aspergillus fumigatus*			
		*Alternaria alternate*			
Squamatic acid	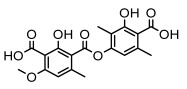	*Staphylococcus aureus*	MIC: 1,250,000	Chloramphenicol (MIC: 5)	[58]
Sekikaic acid	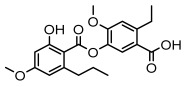	*Staphylococcus aureus*	IR: 50		[62]
		*Streptococcus mutans*	IR: 60		
		*Streptomyces viridochromogenes*	IR: 55		
		*Bacillus subtilis*	IR: 15 MIC: 125	Chloramphenicol (MIC: 7.81) Vancomycin (MIC: 7.81)	[53,62]
		*Escherichia coli*	IR: 78		[62]
Hyperhomosekikaic acid	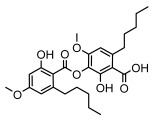	*Bacillus subtilis*	MIC: 125	Chloramphenicol (MIC: 7.81) Vancomycin (MIC: 7.81)	[53]
Lecanorin	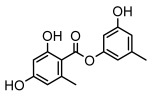	*Candida albicans*	MIC: 64		[65]
Ramalic acid /Obtusatic acid	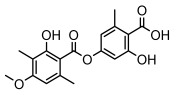	*Staphylococcus aureus*	MIC: 1000	Streptomycin (MIC: 31)	[43]
		*Bacillus cereus*	MIC: 125	Streptomycin (MIC: 16)	
		*Bacillus subtilis*	MIC: 500	Streptomycin (MIC: 16)	
		*Proteus mirabilis* *Escherichia coli*	MIC: 1000	Streptomycin (MIC: 62)	[43]
		*Candida albicans*	MIC: 250	Ketoconazole (MIC: 39)	[43]
		*Cladosporium cladosporioides*	MIC: 500	Ketoconazole (MIC: 39)	
		*Trichoderma viride*		Ketoconazole (MIC: 78)	
		*Penicillium expansum* *Mucor mucedo*	MIC: 1000	Ketoconazole (MIC: 156)	
		*Penicillium chrysogenum Aspergillus niger* *Alternaria alternate* *Fusarium oxysporum*		Ketoconazole (MIC: 78)	
		*Aspergillus flavus*		Ketoconazole (MIC: 312)	
3′-Hydroxyl-5′-propylphenyl 2,4-dihydroxyl-6-methylbenzoate	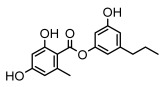	*Aliivibrio fischeri*	IR: 95.5		[79]
Lecanoric acid	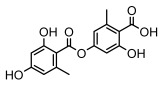	*Clavibacter michiganensis subsp. michiganensis*	MIC > 500	Oxolinic acid (MIC: 31.25) Oxytetracycline (MIC: 125)	[76]
		*Aliivibrio fischeri*	IR: 100		[79]
		*Fusarium fujikuroi*	MIC: 14.8	Amphotericin B (MIC: 3) Isavuconazole (MIC: 5) Natamycin (MIC: 4) Posaconazole (MIC: 0.65) Voriconazole (MIC: 3.7) Fluconazole (MIC: 90) Itraconazole (MIC: 27)	[47]
		*Rhizoctonia solani Kühn*	EC_50_: 35.12		[76]
Olivetoric acid	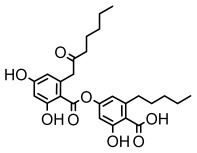	*Bacillus cereus* *Bacillus subtilis* *Staphylococcus aureus*	MIC: 623.48		[70]
		*Listeria monocytogenes*	MIC: 2493.92		
		*Streptococcus faecalis*	MIC: 9999.66		
		*Salmonella Typhimurium*	MIC: 19,975.34		[70]
		*Escherichia coli*			
		*Proteus vulgaris*	MIC: 2493.92		
		*Aeromonas hydrophila*			
		*Yersinia enterocolitica*	MIC: 623.48		
		*Candida albicans* *Candida glabrata*	MIC: 1246.96		[70]
		*Fusarium fujikuroi*	MIC: 1000	Amphotericin B (MIC: 3) Isavuconazole (MIC: 5) Natamycin (MIC: 4) Posaconazole (MIC: 0.65) Voriconazole (MIC: 3.7) Fluconazole (MIC: 90) Itraconazole (MIC: 27)	[47]
3′-Hydroxyl-5′-pentylphenyl 2,4-dihydroxyl-6-methylbenzoate	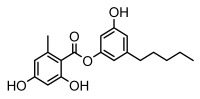	*Aliivibrio fischeri*	IR: 89		[79]
(+)-Erythrin	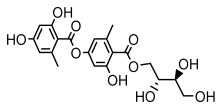	*Streptococcus gordonii*	MIC: 750	Doxycycline (MIC: 0.51)	[64]
		*Porphyromonas gingivalis*	MIC: 375	Doxycycline (MIC: 0.13)	
Gyrophoric acid	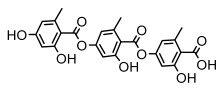	*Bacillus subtilis*	MIC: 19		[62]

MIC: minimum inhibitory concentration; ED_50_: effective dose 50; IZ: inhibition zone diameter; IR: inhibition rate; EC_50_: half maximal effective concentration.

**Table 4:** Antimicrobial activity of depsidones.

Compounds	Structures	Object Strains	Samples	Positive Control	References
			MIC/IC_50_ (µg/mL or µM)/IR/RIZD (%)		
Salazinic acid	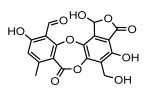	*Bacillus mycoides*	MIC: 0.0008/0.015	Streptomycin (MIC: 7.81)	[59,84]
		*Bacillus subtilis*	MIC: 0.0008/0.0312	Streptomycin (MIC: 7.81)	[59,84]
		*Bacillus cereus*	MIC: 63		[59]
		*Staphylococcus aureus*	MIC: 0.125	Streptomycin (MIC: 15.72)	[84]
		*Mycobacterium smegmatis* MDR-R	MIC: 50	Rifampicin (MIC > 200)	[23]
		*Mycobacterium smegmatis* MDR-40	MIC: 50	Rifampicin (MIC: 100)	[23]
		*Mycobacterium smegmatismegmatis*	MIC: 100	Rifampicin (MIC: 0.2)	[23]
		*Mycobacterium tuberculosis* H37Ra	MIC > 200		
		*Mycobacterium tuberculosis* MDR-A8			
		*Mycobacterium tuberculosis* MDR-V791			
		*Mycolicibacterium aurum*	MIC: 250		[27]
			
		*Penicillium verrucosum*	MIC: 0.5	Ketoconazole (MIC: 3.9)	[84]
		*Klebsiella pneumoniae*	MIC: 0.5	Streptomycin (MIC: 31.25)	[84]
		*Escherichia coli*	MIC: 1	Streptomycin (MIC: 31.25)	[84]
		*Candida albicans*	MIC: 0.25	Ketoconazole (MIC: 1.95)	[84]
		*Aspergillus flavus*	MIC: 1	Ketoconazole (MIC: 3.9)	[84]
		*Aspergillus fumigatus*			
		*Penicillium purpurescens*			
		*Fusarium udum Butler*	IC_50_: 88.20		[59]
Protocetraric acid	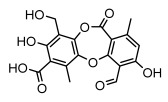	*Bacillus mycoides*	MIC: 0.015/15	Streptomycin (MIC: 7.81)	[59,83,84]
		*Bacillus subtilis*	MIC: 0.015/15/64	Streptomycin (MIC: 7.81)	[59,84]
		*Staphylococcus aureus*	MIC: 0.015/32/12.5/15	Streptomycin (MIC: 15.72)	[59,83,84]
		*Mycobacterium smegmatismegmatis*	MIC: 2	Ciprofloxacin (MIC: 4)	[84]
		*Mycobacterium tuberculosisuberculosis*	MIC: 125		[59]
		*Staphylococcus epidermidis*	MIC: 64	Ciprofloxacin (MIC: 4)	[83]
		*Streptococcus faecalis*	MIC: 64	Ciprofloxacin (MIC: 2)	
		*Vibrio cholerae*	MIC: 2	Ciprofloxacin (MIC: 4)	[83]
		*Proteus vulgaris*	MIC: 4	Ciprofloxacin (MIC: 4)	[83]
		*Escherichia coli*	MIC: 4	Ciprofloxacin (MIC: 2)	
		*Pseudomonas aeruginosa*	MIC: 8	Ciprofloxacin (MIC: 4)	
		*Salmonella typhi*	MIC: 500/0.5		[59,84]
		*Klebsiella pneumoniae*	MIC: 1000/1	Streptomycin (MIC: 31.25)	[83,84]
		*Proteus mirabilis*	MIC: 16	Ciprofloxacin (MIC: 1)	[83]
		*Penicillium purpurescens*	MIC: 1	Ciprofloxacin (MIC: 2) Amphotericin B (MIC: 4)	[83]
		*Fusarium fujikuroi*	MIC: 12.6	Amphotericin B (MIC: 3) Isavuconazole (MIC: 5) Natamycin (MIC: 4) Posaconazole (MIC: 0.65) Voriconazole (MIC: 3.7) Fluconazole (MIC: 90) Itraconazole (MIC: 27)	[47]
		*Penicillium verrucosum*	MIC: 0.5	Ketoconazole (MIC: 3.9)	[84]
		*Candida albicans*	MIC: 64/0.25	Amphotericin B (MIC: 1) Ketoconazole (MIC: 1.95)	[83,84]
		*Campylobacter gastri*	MIC: 64	Amphotericin B (MIC: 1)	[83]
		*Aspergillus flavus*	MIC: 125	Amphotericin B (MIC: 4)	[83]
		*Aspergillus fumigatus*	MIC: 0.25	Ketoconazole (MIC: 3.9)	[84]
		*Candida tropicalis*	MIC: 125	Amphotericin B (MIC: 2)	
		*Candida glabrata*	MIC: 250	Amphotericin B (MIC: 1)	[83]
		*Trichophyton rubrum*	MIC: 1000/1	Amphotericin B (MIC: 4)	[59,83]
Variolaric acid	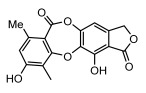	*Streptococcus gordonii*	MIC: 375	Doxycycline (MIC: 0.51)	[64]
		*Porphyromonas gingivalis*		Doxycycline (MIC: 0.13)	
		*Escherichia coli*	IR: 3.2		[59]
Stictic acid	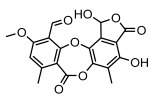	*Francisella tularensis*	IC_50_: 13		[59]
		*Yersinia pestis*	IC_50_: 27		
Norstictic acid	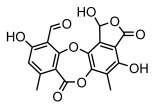	*Fusarium fujikuroi*	MIC: 16.1	Amphotericin B (MIC: 3) Isavuconazole (MIC: 5) Natamycin (MIC: 4) Posaconazole (MIC: 0.65) Voriconazole (MIC: 3.7) Fluconazole (MIC: 90) Itraconazole (MIC: 27)	[47]
Psoromic acid	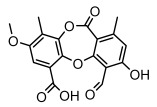	*Bacillus cereus*	MIC: 62.5	Streptomycin (MIC: 4)	[68]
		*Bacillus subtilis*			
		*Mycobacterium tuberculosisuberculosis*	MIC: 3.2~4.1		[82]
		*Mycobacterium tuberculosisuberculosis*	MIC: 62.5		[59]
		*Streptococcus gordonii*	MIC: 11.72	Doxycycline (MIC: 0.51)	[64]
			
		*Staphylococcus epidermidis*	MIC: 125	Streptomycin (MIC: 2)	[68]
		*Staphylococcus aureus*	MIC: 250	Streptomycin (MIC: 2)	
		*Escherichia coli*	MIC: 125 IR: 18.2	Streptomycin (MIC: 4)	[59,68]
		*Shigella sonnei*	MIC: 250	Streptomycin (MIC: 4)	[68]
		*Porphyromonas gingivalis*	MIC: 5.86	Doxycycline (MIC: 0.13)	[64]
Hypoconstictic acid	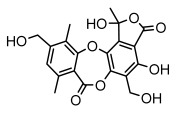	*Staphylococcus aureus*	MIC: 31	Streptomycin (MIC: 2)	[68]
		*Bacillus subtilis*	MIC: 250	Streptomycin (MIC: 4)	
		*Bacillus cereus*			
		*Staphylococcus epidermidis*	MIC > 250	Streptomycin (MIC: 2)	
		*Escherichia coli*	MIC: 62.5	Streptomycin (MIC: 4)	[68]
		*Shigella sonnei*	MIC > 250	Streptomycin (MIC: 4)	
2’-O-Methylhypostictic acid	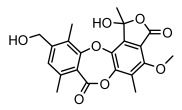	*Bacillus cereus*	MIC: 31	Streptomycin (MIC: 2)	[68]
		*Staphylococcus epidermidis*	MIC: 62.5		
		*Bacillus subtilis*		Streptomycin (MIC: 4)	
Menegazziaic acid	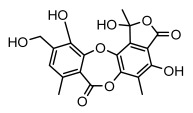	*Staphylococcus aureus*	MIC > 250	Streptomycin (MIC: 2)	[68]
		*Staphylococcus epidermidis*			
		*Bacillus cereus*	MIC: 250	Streptomycin (MIC: 4)	
		*Bacillus subtilis*			
		*Escherichia coli*	MIC: 31	Streptomycin (MIC: 4)	[68]
		*Shigella sonnei*	MIC: 250		
Pannarin	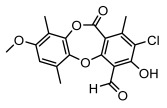	*Methicillin-resistant Staphylococcus aureus*	bactericidal action		[59]
Galbinic acid	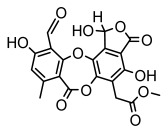	*Bacillus cereus*	MIC: 62.5	Streptomycin (MIC: 4)	[68]
		*Bacillus subtilis*			
		*Staphylococcus aureus*	MIC: 250	Streptomycin (MIC: 2)	
		*Staphylococcus epidermidis*	MIC > 250		
		*Shigella sonnei*	MIC > 250	Streptomycin (MIC: 4)	[68]
		*Escherichia coli*	MIC: 125		
Lobaric acid	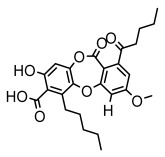	*Staphylococcus aureus-1199B (NorA)*	MIC: 8	Norfloxacin (MIC: 32)	[74]
		*XU212 (TetkmecA)*	MIC: 32	Tetracycline (MIC: 128)	
		*Methicillin-resistant Staphylococcus aureus-16*		Oxacillin (MIC: 512)	
		*RN4220 (MsrA)*		Erythromycin (MIC: 128)	
		*Mycobacterium tuberculosis MDR-A8* *Mycobacterium smegmatis MDR-40*	MIC: 50	Rifampicin (MIC: 100)	[23]
		*Mycobacterium tuberculosis MDR-V791* *Mycobacterium smegmatis MDR-R*		Rifampicin (MIC > 200)	
		*Mycobacterium smegmatismegmatis*		Rifampicin (MIC: 0.2)	
		*Methicillin-resistant Staphylococcus aureus-15*	MIC: 64	Oxacillin (MIC: 32)	[74]
		*Staphylococcus aureus-ATCC 25923*		Norfloxacin (MIC: 32)	
		*Methicillin-resistant Staphylococcus aureus*	MIC: 64	Clindamycin (MIC: 8192) Erythromycin (MIC: 1024) Gentamicin (MIC: 256) Levofloxacin (MIC ≤ 0.5) Oxacillin (MIC: 8)	[75]
		*Mycobacterium tuberculosisH37Ra*	MIC: 100	Rifampicin (MIC: 0.2)	[23]
		*Clavibacter michiganensis subsp. michiganensis*	MIC: 250	Oxolinic acid (MIC: 31.25) Oxytetracycline (MIC: 125)	[76]
		*Streptococcus mutans*	MIC: 20	Penicillin G (MIC: 0.15)	[85]
		*Porphyromonas gingivalis*	MIC: 80	Penicillin G (MIC: 0.29)	
Himantormione A	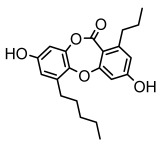	*Staphylococcus aureus*	IC_50_: 3590	Kanamycin (IC_50_: 42)	[69]
Himantormione B	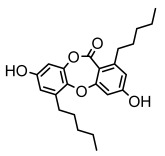	*Staphylococcus aureus*	IC_50_: 701		
α-Collatolic acid	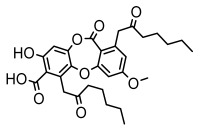	*Methicillin-resistant Staphylococcus aureus*	MIC: 128	Clindamycin (MIC: 8192) Erythromycin (MIC: 1024) Gentamicin (MIC: 256) Levofloxacin (MIC ≤ 0.5) Oxacillin (MIC: 8)	[75]
		*Escherichia coli*	IR: 103.4		[59]
Physodic acid	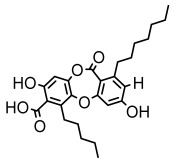	*Bacillus subtilis*	MIC: 0.8 MIC: 6240		[59,70]
		*Bacillus mycoides*	MIC: 1.6		
		*Staphylococcus aureus*	RIZD: 118.78 MIC: 25,000		[29,70]
		*Staphylococcus aureus-1199B (NorA)*	MIC: 16	Norfloxacin (MIC: 32)	[74]
		*Staphylococcus aureus-ATCC 25923*	MIC: 32	Norfloxacin (MIC: 1)	
		*Methicillin-resistant Staphylococcus aureus-15*		Oxacillin (MIC: 32)	
		*Staphylococcus aureus-XU212*		Tetracycline (MIC: 128)	
		*Methicillin-resistant Staphylococcus aureus-16*		Oxacillin (MIC: 512)	
		*Staphylococcus aureus-RN4220*		Erythromycin (MIC: 128)	
		*Bacillus cereus*	MIC: 3120		[70]
		*Listeria monocytogenes*			
		*Streptococcus faecalis*	MIC: 25,000		
		*Proteus vulgaris*	MIC: 25,000		[70]
		*Yersinia enterocolitica*	MIC: 3120		
		*Candida albicans*	MIC: 3120		[70]
		*Candida glabrata*			
3-Hydroxyphysodic acid	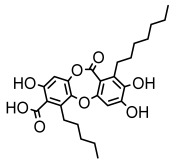	*Staphylococcus aureus-RN4220 (MsrA)*	MIC: 32	Erythromycin (MIC: 128)	[74]
		*Staphylococcus aureus-ATCC 25923*	MIC: 64	Norfloxacin (MIC: 1)	
		*Staphylococcus aureus-1199B (NorA)*		Norfloxacin (MIC: 32)	
		*EMethicillin-resistant Staphylococcus aureus-16*		Oxacillin (MIC: 512)	
		*EMethicillin-resistant Staphylococcus aureus-15*		Oxacillin (MIC: 32)	
		*Staphylococcus aureus-XU212 (Tetk, mecA)*	MIC: 128	Tetracycline (MIC: 128)	
Hypoprotocetraric acid	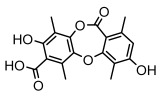	*Streptococcus gordonii*	MIC: 250	Doxycycline (MIC: 0.51)	[64]
		*Porphyromonas gingivalis*	MIC: 62.5	Doxycycline (MIC: 0.13)	
Conhypoprotocetraric acid	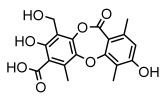	*Streptococcus gordonii*	MIC: 700	Doxycycline (MIC: 0.51)	[64]
		*Porphyromonas gingivalis*	MIC: 175	Doxycycline (MIC: 0.13)	[64]
Fumarprotocetraric acid	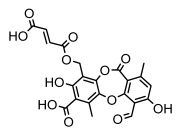	*Bacillus cereus*	MIC: 4.6		[30]
		*Bacillus subtilis*			
		*Listeria monocytogenes*			
		*Streptococcus faecalis*	MIC: 150		
		*Staphylococcus aureus*	MIC: 37.5		
		*Klebsiella pneumoniae*	MIC: 31		[59]
		*Proteus vulgaris*	MIC: 37.5		[30]
		*Aeromonas hydrophila*	MIC: 150		
		*Candida albicans*	MIC: 18.7		[30]
		*Candida glabrata*			

MIC: minimum inhibitory concentration; IR: inhibition rate; RIZD (%): relative inhibition zone diameter; IC_50_: half maximal inhibitory concentration.

**Table 5:** Antimicrobial activity of dibenzofuran compounds.

Compounds	Structures	Object Strains	Samples	Positive Control	References
			MIC/IC_50_ (µg/mL or µM)/IZ (mm)/BEC (µg/mL)		
Usnic acid	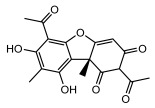	*Staphylococcus aureus*	MIC: 7.81, 1.0, 21, 0.15, 156	Chloramphenicol (MIC: 31.25) Vancomycin (MIC: 15.63) Streptomycin (MIC: 15.72) Tetracycline (MIC < 0.06) Ampicillin (MIC < 0.06)	[20,38,53,84,93]
		*Methicillin-susceptible Staphylococcus aureus (Sa3,Sa13)*	MIC: 2		[81]
		*Methicillin-resistant Staphylococcus aureus (Sa1, Sa10,Sa14, Sa15)*	MIC: 8		
		*Methicillin-resistant Staphylococcus aureus*	MIC: 25~50		[20]
		*Bacillus subtilis*	IZ: 15.0~21.0, 7.81, 0.5 MIC: 0.0008	Chloramphenicol (MIC: 7.81) Vancomycin (MIC: 7.81) Streptomycin (MIC: 7.81)	[20,21,53,84]
		*Bacillus cereus*	IZ: 23.7	Chloramphenicol (IZ: 22.3)	[94]
		*Bacillus mycoides*	MIC: 0.0008	Streptomycin (MIC: 7.81)	[84]
		*Bacillus megaterium*	IZ: 17.0~22.0		[21]
		*Enterococcus casseliflavus*	IZ: 19.7	Levofloxacin (IZ: 25.0) Tetracycline (IZ: 26.0)	[22]
		*Streptococcus pyogenes*	IZ: 12.0	Levofloxacin (IZ: 21.0) Tetracycline (IZ: 27.0)	[22]
		*Streptococcus pneumoniae*	IZ: 17.0, 17.3	Levofloxacin (IZ: 22.0) Tetracycline (IZ: 30.7) Ofloxacin (IZ: 19.3) Ceftriaxone (IZ: 21.0)	[22,49]
		*Mycobacterium abscessus ATCC 19977*	MIC: 18.15	Amikacin (MIC: 1.71) Ciprofloxacin (MIC: 3.02) Clarithromycin (MIC: 0.67)	[88]
		*Mycobacterium abscessus AT07*	MIC: 9.07	Amikacin (MIC: 3.41) Ciprofloxacin (MIC: 6.03) Clarithromycin (MIC: 0.17)	[88]
		*Mycobacterium abscessus AT46*		Amikacin (MIC: 1.71) Ciprofloxacin (MIC: 12.07) Clarithromycin (MIC: 0.33)	
		*Mycobacterium abscessus AT52*		Amikacin (MIC: 6.83) Ciprofloxacin (MIC: 24.14) Clarithromycin (MIC: 171.13)	
		*Mycobacterium tuberculosis H37Ra*	MIC: 50	Rifampicin (MIC: 0.2)	[23]
		*Mycobacterium tuberculosis MDR-A8*	MIC: 25	Rifampicin (MIC: 100)	
		*Mycobacterium tuberculosis MDR-V791 Mycobacterium smegmatismegmatis MDR-R*	MIC: 12.5	Rifampicin (MIC > 200)	
		*Mycobacterium smegmatismegmatis*	MIC: 12.5	Rifampicin (MIC: 0.2)	
		*Mycobacterium smegmatismegmatis MDR-40*	MIC: 12.5	Rifampicin (MIC: 100)	
		*Clavibacter michiganensis subsp. michiganensis*	MIC: 7.812	Oxolinic acid (MIC: 31.25) Oxytetracycline (MIC: 125)	[76]
		*Pseudomonas aeruginosa*	IZ: 16.7 MIC: 133	Ofloxacin (IZ: 19.3) Ceftriaxone (IZ: 21.0)	[38,49]
		*Escherichia coli*	MIC: 20, 0.25, 225	Streptomycin (MIC: 31.25)	[20,38,84]
		*Escherichia coli*	IZ: 7.0, 18.6, 16	Levofloxacin (IZ: 31.0) Tetracycline (IZ: 21.0) Chloramphenicol (IZ: 23.2) Ampicillin (IZ: 21.0)	[22,37,94]
		*Klebsiella pneumoniae*	MIC: 0.0625 IZ: 11.3	Streptomycin (MIC: 31.25) Chloramphenicol (IZ: 17.5)	[84,94]
		*Proteus mirabilis*	MIC < 10,000	Tetracycline (MIC > 128) Ampicillin (MIC > 128)	[93]
		*Salmonella typhi*	IZ: 14.0, 18.1	Ampicillin (IZ: 17.0) Chloramphenicol (IZ: 23.8)	[37,94]
		*Salmonella enterica*	MIC < 10,000	Tetracycline (MIC: 2) Ampicillin (MIC: 1)	[93]
		*Salmonella typhimurium*		Tetracycline (MIC: 2) Ampicillin (MIC: 2)	
		*Vibrio harveyi*	MIC: 20		[20]
		*Fusarium fujikuroi*	MIC: 18.6	Amphotericin B (MIC: 3) Isavuconazole (MIC: 5) Natamycin (MIC: 4) Posaconazole (MIC: 0.65) Voriconazole (MIC: 3.7) Fluconazole (MIC: 90) Itraconazole (MIC: 27)	[47]
		*Achlya bisexualis*	MIC: 8		[46]
		*Pythium sp.*			
		*Saprolegnia parasitica*	MIC: 2		
		*Aspergillus flavus*	MIC: 0.5	Ketoconazole (MIC: 3.9)	[84]
		*Aspergillus fumigatus*	MIC: 0.125	Ketoconazole (MIC: 3.9)	
		*Aspergillus niger*	MIC: 10	Amphotericin B (MIC: 0.98) Fluconazole (MIC: 250)	
		*Penicillium purpurescens*	MIC: 0.5	Ketoconazole (MIC: 3.9)	[84]
		*Penicillium verrucosum*			[93]
		*Candida albicans*	MIC: 0.25	Ketoconazole (MIC: 1.95)	[84]
		*Saccharomyces cerevisiae*	MIC: 5	Fluconazole (MIC: 7.81)	[93]
		*Malassezia*	IZ: 20.0		[95]
(+)-Usnic acid	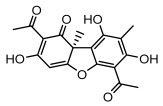	*Staphylococcus aureus*	MIC: 7.5, 12.5, IZ: 20.0	Vancomycin (MIC: 1.47)	[87,91,96]
		*Staphylococcus aureus-ATCC 25923*	MIC: 16	Norfloxacin (MIC: 1)	[74]
		*Staphylococcus aureus XU212 (Tetk, mecA)*		Tetracycline (MIC: 128)	
		*Staphylococcus aureus RN4220 (MsrA)*	MIC: 8	Erythromycin (MIC: 128)	[74]
		*Staphylococcus aureus-1199B (NorA)*		Norfloxacin (MIC: 32)	
		*Methicillin-resistant Staphylococcus aureus-15*	MIC: 16	Oxacillin (MIC: 32)	[74]
		*Methicillin-resistant Staphylococcus aureus-16*		Oxacillin (MIC: 512)	
		*Staphylococcus epidermidis*	MIC: 3.12	Vancomycin (MIC: 2.95)	[91]
		*Staphylococcus haemolyticus*	MIC: 12.5	Vancomycin (MIC: 2.95)	
		*Staphylococcus haemolyticus*	MIC: 25		[87]
		*Bacillus subtilis*	MIC: 8	Streptomycin (MIC: 4)	[68]
		*Bacillus cereus*			
		*Mycobacterium avium*	MIC: 16	Isoniazid (MIC: 1.0)	[24]
		*Mycobacterium tuberculosisuberculosis*	MIC: 8 IZ: 8	Isoniazid (MIC: 0.03)	[24,87]
		*Mycobacterium kansasii*	MIC: 8	Isoniazid (MIC: 0.05)	[24]
		*Enterococcus faecium*	MIC > 50, 6.25		[44,87]
		*Escherichia coli*	MIC: 31		[68]
		*Helicobacter pylori*	MIC: 4~8		[87]
		*Candida albicans*	MIC: 64		[65]
		*Candida orthopsilosis*	BEC_50_: 3.9 BEC_80_: 31.25		[87]
		*Candida parapsilosis*	BEC_50_: 3.9 BEC_80_: 62.5		
(−)-Usnic acid	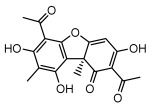	*Staphylococcus aureus*	IZ: 25~40 MIC: 100		[87]
		*Staphylococcus aureus*	MIC: 2.4, 2.5, 7.5	Chloramphenicol (MIC: 5)	[30,57,58]
		*Methicillin-resistant Staphylococcus aureus*	MIC: 2.5~7.5		[58]
		*Methicillin-resistant Staphylococcus aureus (Cl)*	MIC: 25~50 32~128	Oxacillin (MIC: 0.078) Oxacillin (MIC: 16–128)	[97,98]
		*Bacillus cereus*	MIC: 0.15		[30]
		*Bacillus subtilis*	MIC: 0.61		
		*Streptococcus faecalis*	MIC: 0.15		
		*Listeria monocytogenes*	MIC: 0.31		[30]
		*Proteus vulgaris*	MIC: 0.15		
		*Aeromonas hydrophila*	MIC: 1.2		
		*Candida albicans*	MIC: 0.15		[30]
		*Candida glabrata*			
Usenamine E	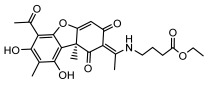	*Candida albicans*	MIC: 64		[65]
Usenamine F	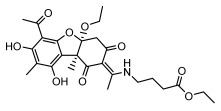	*Candida albicans*	MIC: 64		[65]
Usenamine G	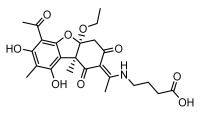	*Candida albicans*	MIC: 64		[65]
Usenamine H	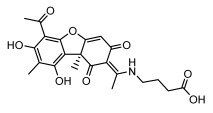	*Candida albicans*	MIC: 64		[65]
Isousone	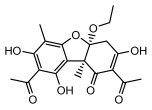	*Trichophyton rubrum* spp.	MIC: 41		[92]
Usone	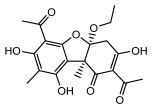	*Trichophyton rubrum* spp.	MIC: 41		[92]
Perfluorophenacyl	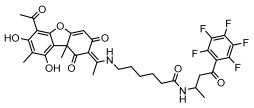	*Bacillus subtilis*	IZ: 12.0 MIC: 158.1	Streptomycin (IZ: 33.0 MIC: 3)	[90]
1, 3, 7, 9-Tetrahydroxy-2, 8-dimethyl-4, 6-di (ethanoyl) dibenzofuran	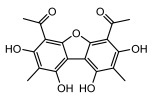	*Escherichia coli*	IC_50_: 18		[99]
2,6-Difluorophenyl	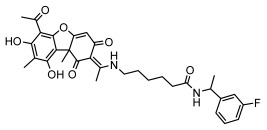	*Salmonella Typhi*	MIC: 11		[90]
		*Bacillus subtilis*		Streptomycin (IZ: 33.0 MIC: 3)	
		*Escherichia coli*	MIC: 6	Streptomycin (IZ: 37.0 MIC: 3)	
2-Acylnaphthalenyl	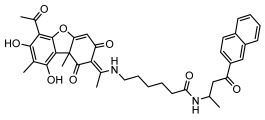	*Mtb H37Rv*	MIC: 5.3		[89]
3,4-Difluorophenacyl	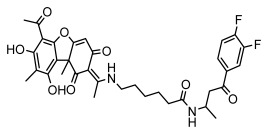	*Bacillus subtilis*	IZ: 12.0 MIC: 172.8	Penicillin (IZ: 28.0 MIC: 3.5) Streptomycin (IZ: 24.0 MIC: 8.1)	[89]
		*Mtb H37Rv*	MIC: 5.4		
N-Acylmorpholinyl	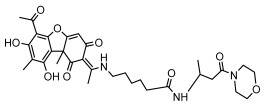	*Bacillus subtilis*	IZ: 12.0 MIC: 90.7	Penicillin (IZ: 28.0 MIC: 3.5) Streptomycin (IZ: 24.0 MIC: 8.1)	[89]
Didymic acid	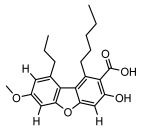	*Staphylococcus aureus*	MIC: 7.5		[57]
Condidymic acid	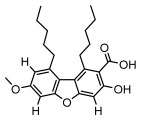	*Staphylococcus aureus*	MIC: 7.5		[57]
3-Fluoro-5-trifluoromethylphenyl	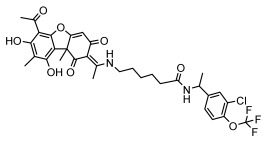	*Salmonella Typhi*	MIC: 10		[90]
Hexanoic acid	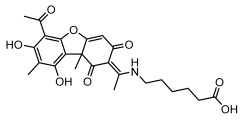	*Bacillus subtilis*	MIC: 3	Streptomycin (IZ: 33.0 MIC: 3)	[90]
		*Streptococcus mutans*	MIC: 7		
		*Salmonella Typhi*	MIC: 3		[90]

MIC: minimum inhibitory concentration; IZ: inhibition zone diameter; BEC50: biofilm-eradicating concentration 50; IC_50_: half maximal inhibitory concentration.

**Table 6:** Antimicrobial activity of other phenol derivatives.

Compounds	Structures	Object Strains	Samples	Positive Control	References
			MIC (µg/mL)/IZ (mm)		
3-Chloro-4,6-dimethoxy-1-methyl-9H-xanthen-9-one	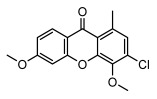	*Staphylococcus aureus*	IZ: 9.5		[102]
		*Enterococcus faecium*	IZ: 10.0		
2,7-Dichloro-3,4,6-trimethoxy-1-methyl-9H-xanthen-9-one	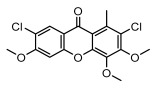	*Epidermophyton floccosum*	MIC: 4		[102]
		*Trichophyton rubrum Microsporum canis*	MIC: 8		
Lepraric acid	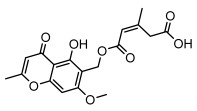	*Streptococcus gordonii*	MIC > 2500	Doxycycline (MIC: 0.51)	[64]
		*Porphyromonas gingivalis*	MIC: 625	Doxycycline (MIC: 0.13)	
Eumitrin F	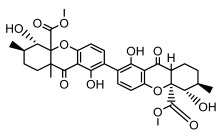	*Bacillus subtilis*	MIC: 62.5		[103]
		*Escherichia coli*	MIC: 62.5		[103]
Eumitrin G	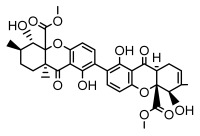	*Bacillus subtilis*	MIC: 62.5		[103]
		*Escherichia coli*	MIC: 62.5		[103]
Eumitrin H	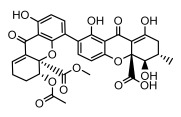	*Bacillus subtilis*	MIC: 62.5		[103]
		*Escherichia coli*	MIC: 62.5		[103]
Hybocarpone	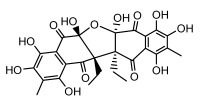	*Staphylococcus aureus*	MIC: 4	Norfloxacin (MIC: 1)	[74]
		*Staphylococcus aureus-RN4220 (MsrA)*		Erythromycin (MIC: 128)	
		*Methicillin-resistant Staphylococcus aureus-15*		Oxacillin (MIC: 32)	
		*Methicillin-resistant Staphylococcus aureus-16*	MIC: 8	Oxacillin (MIC: 512)	
		*Staphylococcus aureus-1199B (NorA)*		Norfloxacin (MIC: 32)	
		*Staphylococcus aureus-XU212 (Tetk, mecA)*		Tetracycline (MIC: 128)	

MIC: minimum inhibitory concentration; IZ: inhibition zone diameter.

**Table 7:** Antimicrobial activity of higher fatty acids and esters compounds.

Compounds	Structures	Object Strains	Samples	Positive Control	References
			MIC/ED_50_ (µg/mL)/IZ (mm)		
Protolichesterinic acid	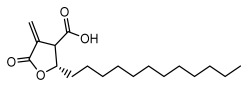	Methicillin-resistant *Staphylococcus aureus*	MIC: 64	Clindamycin (MIC: 8192) Erythromycin (MIC: 1024) Gentamicin (MIC: 256) Levofloxacin (MIC ≤ 0.5) Oxacillin (MIC: 8)	[75]
		*Pythium debaryanum*	ED_50_: 16.07	Hexaconazole (ED_50_: 25.92)	[27]
		*Rhizoctonia solani*	ED_50_: 23.09		
Constipatic acid	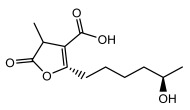	*Candida albicans*	MIC: 64		[65]
Lichesterinic acid B-10	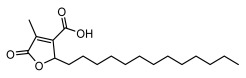	*Porphyromonas gingivalis*	MIC: 0.073	Doxycycline (MIC: 0.13)	[104]
Lichesterinic acid B-7		*Porphyromonas gingivalis*	MIC: 75		
Lichesterinic acid B-12		*Porphyromonas gingivalis*	MIC: 0.037		
Lichesterinic acid B-13		*Porphyromonas gingivalis*	MIC: 0.293		
18R-hydroxy-dihydroalloprotolichesterinic acid	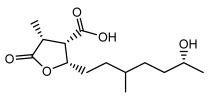	*Candida albicans*	MIC: 64		[65]

MIC: minimum inhibitory concentration; ED_50_: effective dose 50; IZ: inhibition zone diameter.

**Table 8:** Antimicrobial activity of other categories of lichens substances.

Compounds	Structures	Object Strains	Samples	Positive Control Antibiotics	References
			MIC (µg/mL or µM)/IC_50_/EC_50_ (µg/mL)/IZ (mm)		
Rhizocarpic acid	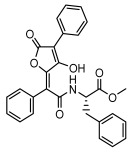	*Bacillus subtilis*	MIC: 50	Tetracycline (MIC: 6.3)	[105]
		*Staphylococcus aureus*	MIC: 32	Norfloxacin (MIC: 1)	[74]
		*RN4220 (MsrA)*		Erythromycin (MIC: 128)	
		*Methicillin-resistant Staphylococcus aureus-15*		Oxacillin (MIC: 32)	
		*Methicillin-resistant Staphylococcus aureus-16*		Oxacillin (MIC: 512)	
		*Staphylococcus aureus-1199B (NorA)*	MIC: 64	Norfloxacin (MIC: 32)	
		*XU212 (Tetk, mecA)*		Tetracycline (MIC: 128)	
7-Hydroxy-3-(2-methylbut-3-en2-yl)-chromen-2-one	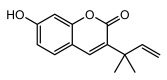	*Bacillus subtilis*	MIC: 2620		[107]
		*Klebsiella pneumoniae*	MIC: 1290		
		*Escherichia coli Pseudomonas aeruginosa*	MIC: 1560		
		*Candida albicans*	MIC: 6250		
		*Aspergillus fumigatus*	MIC: 7250		
Stereocalpin A	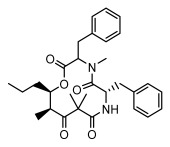	*Escherichia coli*	IC_50_: 28		[99]
Stereocalpin B	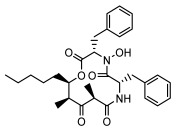	*Escherichia coli*	IC_50_: 30		[99]
Epiforellic acid	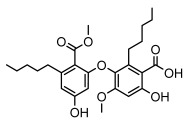	*Methicillin-resistant Staphylococcus aureus*	MIC: 32	Clindamycin (MIC: 8192) Erythromycin (MIC: 1024) Gentamicin (MIC: 256) Levofloxacin (MIC ≤ 0.5) Oxacillin (MIC: 8)	[75]
Cryptothecin A	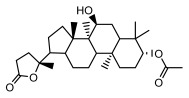	*Candida albicans*	Weak activity		[108]
Vulpinic acid	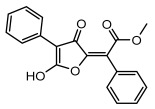	*Methicillin-resistant Staphylococcus aureus*	MIC: 31,250		[106]
		*Streptococcus gordonii*	MIC: 187.5	Doxycycline (MIC: 0.51)	[64]
		*Clavibacter michiganensis subsp. michiganensis*	MIC: 3.9	Oxolinic acid (MIC: 31.25) Oxytetracycline (MIC: 125)	[76]
		*Porphyromonas gingivalis*	MIC: 375	Doxycycline (MIC: 0.13)	[64]
		*Sclerotinia sclerotiorum*	EC_50_: 2.8		[76]
Uridine	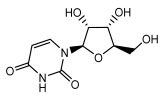	*Escherichia coli*	IZ: 6.3	Gentamicin (IZ: 12.4)	[65]
4-(Acylamino)butyramides	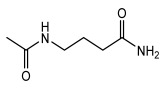	*Candida albicans*	MIC: 64		[65]
(+)-Roccellic acid	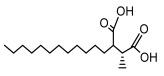	*Streptococcus gordonii*	MIC: 46.9	Doxycycline (MIC: 0.51)	[64]
		*Porphyromonas gingivalis*		Doxycycline (MIC: 0.13)	
Caperatic acid	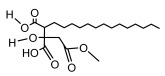	*Staphylococcus aureus*	MIC: 10		[96]

MIC: minimum inhibitory concentration; IZ: inhibition zone diameter; EC_50_: half maximal effective concentration; IC_50_: half maximal inhibitory concentration.

**Table 9:** Antiviral activity of lichen-derived compounds.

Compounds	Structures	Object Strains	Samples	Positive Control Antibiotics	References
			IC_50_/ED_50_ (µg/mL or µM)/IZ (mm)/IR (%)/SI		
Methyl *β*-orcinol-carboxylate	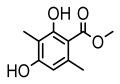	Hepatitis C Virus	IC_50_: 50.6		[111]
Atranol	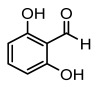	Hepatitis C Virus	IC_50_: 40.3	Telaprevir (IC_50_: 0.18) Erlotinib (IC_50_: 0.64)	
Methyl orsellinate	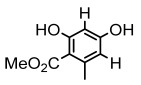	Hepatitis C Virus	IC_50_ > 100		
Barbatic acid	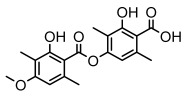	Epstein–Barr virus	IC_50_ > 100		[62]
Diffractic acid	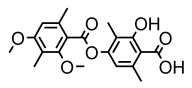	Epstein–Barr virus	IC_50_: >100		[62]
Evernic acid	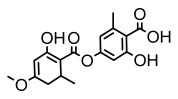	Epstein–Barr virus	IR: 64.6		[62]
Atranorin	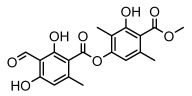	Hepatitis C virus	IC_50_: 22.3 SI > 4.5	Telaprevir (IC_50_: 0.18) Erlotinib (IC_50_: 0.64)	[111]
Sekikaic acid	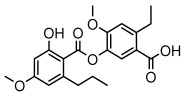	Respiratory syncytial virus rg	IC_50_: 5.69 SI: 5.46		[62,112]
		Respiratory syncytial virus A2	IC_50_: 7.7		
Psoromic acid	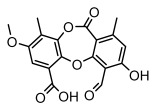	Herpes Simplex Virus Type 1	IC_50_: 1.9 SI: 163.2	Acyclovir (IC_50_: 2.6 SI: 119.2)	[113]
		Herpes Simplex Virus Type 2	IC_50_: 2.7 SI: 114.8	Acyclovir (IC_50_: 2.8 SI: 110.7)	
Usnic acid	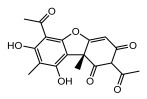	Herpes simplex type 1 virus	IZ > 4		[20]
		Polio type 1 virus	IZ > 4		
(+)-Usnic acid	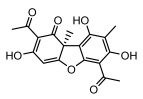	Severe acute respiratory Syndrome Coronavirus 2	IC_50_: 7.99 SI: 6.26	Chloroquine (IC_50_: 6.16 SI: 13.07) Remdesivir (IC_50_: 2.25 SI: 4.24) Lopinavir (IC_50_: 10.8 SI: 6.74)	[115]
		Severe acute respiratory Syndrome Coronavirus 2 Alpha (B.1.1.7)	IC_50_: 6.05 SI: 5.8	Chloroquine (IC_50_: 2.64) Remdesivir (IC_50_: 1.47) Lopinavir (IC_50_: 11.8)	
		Severe acute respiratory Syndrome Coronavirus 2 Beta (B.1.351)	IC_50_: 2.92 SI: 11.1	Chloroquine (IC_50_: 6.22) Remdesivir (IC_50_: 6.48) Lopinavir (IC_50_: 15.3)	
		A(H1N1)pdm09 influenza virus	ED_50_: 51.7 SI: 5.9		[87,116]
		Epstein–Barr virus activation	ED_50_: 1.0		
(−)-Usnic acid	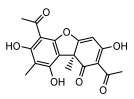	A(H1N1)pdm09 influenza virus	ED_50_: 14.5 SI: 14.4		[87,116]
		Epstein–Barr virus	ED_50_: 5.0		

ED_50_: effective dose 50; IZ: inhibition zone diameter; IR: inhibition rate; IC_50_: half maximal inhibitory concentration; SI: selection index.
